# A combined risk model for the multi-encompassing identification of heterogeneities of prognoses, biological pathway variations and immune states for sepsis patients

**DOI:** 10.1186/s12871-021-01552-x

**Published:** 2022-01-07

**Authors:** Zong-xiu Yin, Chun-yan Xing, Guan-hua Li, Long-bin Pang, Jing Wang, Jing Pan, Rui Zang, Shi Zhang

**Affiliations:** 1 Department of Pulmonary and Critical Care Medicine, Jinan Central Hospital Affiliated to Shandong First Medical University and Shandong Academy of Medical Sciences, Cheeloo College of Medicine, Shandong University, No. 105 Jiefang Road, Jinan, 250013 Shandong Province China; 2 Department of Pulmonary and Critical Care Medicine, Jinan Central Hospital Affiliated to Shandong First Medical University, Jinan, China

**Keywords:** Sepsis, Heterogeneity, Prediction model, Signal transduction pathway, Immune dysfunction

## Abstract

**Background:**

Sepsis is a highly heterogeneous syndrome with stratified severity levels and immune states. Even in patients with similar clinical appearances, the underlying signal transduction pathways are significantly different. To identify the heterogeneities of sepsis from multiple angles, we aimed to establish a combined risk model including the molecular risk score for rapid mortality prediction, pathway risk score for the identification of biological pathway variations, and immunity risk score for guidance with immune-modulation therapy.

**Methods:**

We systematically searched and screened the mRNA expression profiles of patients with sepsis in the Gene Expression Omnibus public database. The screened datasets were divided into a training cohort and a validation cohort. In the training cohort, authentic prognostic predictor characteristics (differentially expressed mRNAs, pathway activity variations and immune cells) were screened for model construction through bioinformatics analysis and univariate Cox regression, and a *P* value less than 0.05 of univariate Cox regression on 28-day mortality was set as the cut-off value. The combined risk model was finally established by the decision tree algorithm. In the validation cohort, the model performance was assessed and validated by C statistics and the area under the receiver operating characteristic curve (AUC). Additionally, the current models were further compared in clinical value with traditional indicators, including procalcitonin (PCT) and interleukin-8 (IL-8).

**Results:**

Datasets from two sepsis cohort studies with a total of 585 consecutive sepsis patients admitted to two intensive care units were downloaded as the training cohort (*n* = 479) and external validation cohort (*n* = 106). In the training cohort, 15 molecules, 20 pathways and 4 immune cells were eventually enrolled in model construction. These prognostic factors mainly reflected hypoxia, cellular injury, metabolic disorders and immune dysregulation in sepsis patients. In the validation cohort, the AUCs of the molecular model, pathway model, immune model, and combined model were 0.81, 0.82, 0.62 and 0.873, respectively. The AUCs of the traditional biomarkers (PCT and IL-8) were 0.565 and 0.585, respectively. The survival analysis indicated that patients in the high-risk group identified by models in the current study had a poor prognosis (*P* < 0.05). The above results indicated that the models in this study are all superior to the traditional biomarkers for the predicting the prognosis of sepsis patients. Furthermore, the current study provides some therapeutic recommendations for patients with high risk scores identified by the three submodels.

**Conclusions:**

In summary, the present study provides opportunities for bedside tests that could quantitatively and rapidly measure heterogeneous prognosis, underlying biological pathway variations and immune dysfunction in sepsis patients. Further therapeutic recommendations for patients with high risk scores could improve the therapeutic system for sepsis.

**Supplementary Information:**

The online version contains supplementary material available at 10.1186/s12871-021-01552-x.

## Introduction

Sepsis is a heterogeneous syndrome with an uncontrolled systemic inflammatory host response to infection which furthermore induces perpetuation of organ dysfunction [1]. Prolonged immune dysfunction induced imbalance between hyper-inflammation and anti-inflammation is identified as main reason of organ dysfunction and lethality in sepsis. Therapeutic ways for sepsis have been limited for decades and the mortality of sepsis is 10–35% [2, 3].

It is generally believed that previous failure of proposed treatments for septic patients is due to substantial heterogeneity in sepsis and the lack of methods to precisely classify patients at molecular biology level [4–6]. The heterogeneity in sepsis complicated the screening specific population who might benefit from adjuvant treatments. The heterogeneity of immune alterations in sepsis actually limited the efficacy and progress of immune modulation [7, 8]. A rapid tool to quantitatively measure immune disorders could potentially classify septic population for accurate immune modulation. Therefore, Burnham et al. [9] stratified patients with sepsis to the four endotypes, and Sweeney et al. [4] built a molecular model to predict mortality for patients with sepsis according to transcriptomic data.

Nevertheless, these landmark studies for explore heterogeneity in sepsis still could not identify heterogeneity at pathogenesis level, therefore fail to achieve more-precise personalized interventions especially pathogenesis-related therapy. This is due to that even in patients with similar clinical surfaces, underlying pathogenesis may be significant different [10, 11]. Obviously, it is robustly believed that better insight into heterogeneity of pathogenesis is crucial to improve precise therapeutic opinions [12–14].

Under this background, we conducted a secondary analysis on high-throughput data of sepsis downloaded from a public database to construct a combined risk model including the molecular risk score, pathway risk score and immunity risk score for better insight into the heterogeneity of sepsis. The molecular risk score, as a rapid assay, could promote accuracy in sepsis prognosis for the appropriate matching of patients with resources. The pathway risk score could indicate heterogeneity of pathogenesis for the further selection of specific adjuvant therapy. The immune risk score could provide a quantitative measure of immune dysfunction for the guidance of immune modulation therapy.

## Methods

**Fig. 1:**
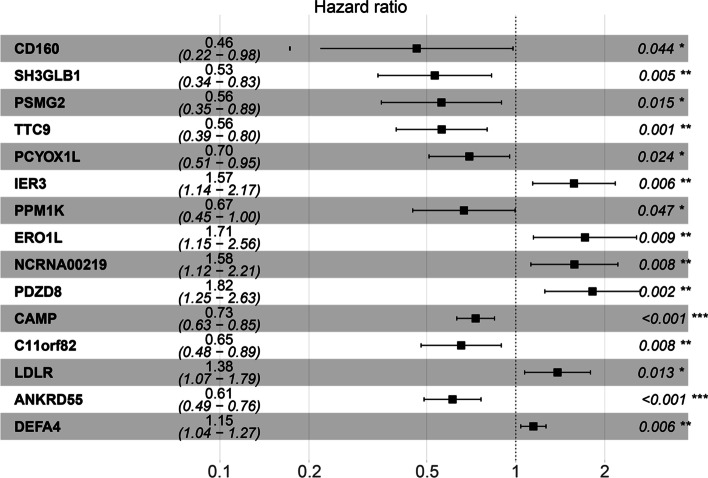
The Molecule-risk model. To construct the Molecule-risk model, two steps (molecular characteristics screening and model establishing) were conducted. Molecular characteristics screening: 15 prognostic molecules were finally enrolled into the Molecule-risk model after univariate Cox regression analyses, Lasso regression analyses and both backward and forward stepwises selection based on the AIC. Molecule-risk model establishing: The final molecular model was constructed by multivariate Cox regression analyses and generated a molecule risk score for every patient. The forest plots of 15 molecules in multivariate Cox regression analyses. The first column was the name of 15 molecules; the second column showed the sample size; the third column illuminated the HR and 95%CI of 15 molecules; the forth column was the forest plots of HR and 95%CI; the last column indicated the *P* value of 15 molecules in multivariate Cox regression analyses

This study included the training cohort to construct the combined risk model and the validation cohort to compare the models’ performance with classical septic indicators such as procalcitonin (PCT) and interleukin-8 (IL-8). The analysis flow plot is shown in Supplemental Material (SM) Fig. 1.

### Database search and study selection

The data discussed in this publication were down-loaded from National Center for Biotechnology Information’s (NCBI’s) Gene Expression Omnibus(GEO) [15, 16]. GEO is an international public repository that archives and freely distributes microarray, next-generation sequencing, and other forms of high-throughput functional genomics data submitted by the research community [15, 16].

The GEO database was searched for all expression microarrays that matched terms associated with sepsis. The datasets were collected from clinical studies investigating sepsis in adults using peripheral blood within 48 h after ICU admission. The exclusion criteria were as follows: (1) studies with septic animal or cell models and (2) studies with a lack of prognostic information. The dataset with the maximum sample size was set as the training cohort, and another dataset was set as the external validation cohort.

All datasets were downloaded as txt files, and outputs from the mRNA array were normal-exponential background corrected and then between-array quantile normalized using the limma R package.

### Statistical analysis

The statistical analysis included 3 steps: (1) the identification of prognostic molecules, pathways and immune cells; (2) the construction of prediction models; and (3) model evaluation.

1. Identification of prognostic molecules, pathways and immune cells:

For assessment underlying pathway activity variation in the sepsis samples, we utilized GSVA algorithm and canonical pathways signature, which could transform the gene by sample matrix into canonical pathways enrichment scores by sample matrix through GSVA R package [17, 18].

To define percentages of immune cells in the sepsis whole blood, CIBERSORT algorithm were conducted. In brief, CIBERSORT algorithm could identify and qualify the absolute fractions of 22 human immune cell phenotypes from transcriptomic data consist of diverse T cell types, B cells, NK cells, and myeloid subsets. The expressions of mRNA, pathways and immune cells were finally enrolled in prognostic features screening [19–21].

To determine prognostic signatures, the univariate Cox proportional hazards model with Bonferroni correction for multiple comparisons was performed on training cohort, with a cut-off value of *P* < 0.05, using the survival R package [22–24]. Furthermore, for reducing overfitting problems, the least absolute shrinkage and selection operator (LASSO) and both backward and forward stepwise selection with the Akaike information criterion (AIC) were utilized to screen the final characteristics through the glmnet R and survival R packages [25, 26].

(2) Construction of the prediction models:

Construction of the sub-models: In the training dataset, the associations of relevant characteristics with survival were evaluated based on Cox proportional hazards regression models. Hazard ratios (HRs) were shown with their 95% CIs. The selected characteristics were incorporated in the nomograms (risk model) to predict the probability of 28-day mortality using the rms R package [27, 28]. Every patient’s risk score was calculated based on the predictive model, and the respective medians of the two groups were set as the cut-off value to divide the patients into high-risk and low-risk groups.

Construction of the combined-model: The risk scores, age, male sex and pneumonia status were assessed by multivariable Cox proportional hazards regression, and the variables that were significantly associated with survival were included in the combined risk model.

To simplify the model for rapid clinical application, the decision tree algorithm was utilized to further optimize the combined risk model [29].

(3) Model evaluation:

In the validation dataset, model performance was assessed according to the discrimination ability and calibration ability. Discriminating ability was evaluated using C statistics and the area under the receiver operating characteristic curve (AUC). Calibration of the nomograms was assessed using chi-square tests, comparing the 28-day mortality of low-risk and high-risk septic populations. In addition, the current models were further compared in clinical value with traditional indicators, including PCT and IL-8.

R × 64 4.0.3 was utilized to conduct all analyses.

## Results

### Patients

After the search strategy, two mRNA datasets (GSE65682 and GSE63042) of patients with sepsis from the GEO public database (585 septic patients) were finally enrolled in the current study [9, 27]. Sepsis was defined according to the Sepsis 1.0 criteria in these two datasets [30, 31]. The patients with sepsis were all enrolled from the ICU.

The septic shock ratio in the training and validation cohorts was 34.8 and 31.1%, respectively. Details of the demographic details are shown in Table 1. The GSE65682 dataset was set as the training cohort, and the GSE63042 dataset was set as the external validation cohort.

**Table 1:** Demographic and clinical characteristics

	GSE65682 Patients with sepsis: 479	GSE63042 Patients with sepsis: 106
Male sex	272(56.8%)	63(59.4%)
Age	63(18–89)	59(38–85)
Country	Netherlands	USA
Pneumonia diagnoses	183(38.0%)	24(22.6%)
Septic shock	167(34.8%)	33(31.1%)
28 day mortality	115(24.0%)	28(26.4%)
APACHE	85 (69–103)^a^	16(9–28)^b^
Main study	Classification for sepsis through transcriptomic data	Bioinformatic analysis for host response in sepsis

Definition of abbreviations: *N* number, *APACHE* Acute Physiology and Chronic Health Evaluation, ^a^APACHE IV; ^b^APACHE II

The dataset of the training cohort was uploaded by Scicluna et al. from the University Medical Center in Utrecht and the Academic Medical Center in Amsterdam. This dataset included 479 patients with sepsis and relatively complete prognostic data. In addition, 42 healthy participants (median age 35 years [IQR 30–63]; 24 [57%] of the 42 participants were men) were also enrolled in the GSE65682 dataset.

GSE63042 data were uploaded by Langley et al. from the Immunology Department at the University of New Mexico, which included 106 septic patients with 28-day mortality information.

### Molecular risk model

A total of 1178 differentially expressed mRNAs among 479 patients with sepsis and 42 healthy participants were identified and screened, as shown in the volcano plot (SM Fig. 2). Furthermore, 359 molecules were further identified as prognostic molecules in sepsis and were significantly associated with 28-day cumulative mortality (*P* < 0.05), as shown in SM Table 1. To construct the optimal model, 15 prognostic molecules were screened into the final molecular model construction. The details of the 15 molecules are shown in Fig. 1, and the screening process is shown in SM Table 2 and SM Fig. 3. The main functions of these prognostic molecules included immune response, cell apoptosis and protein synthesis in sepsis.

Based on these 15 prognostic molecules, a molecular model and nomograms were constructed, as shown in SM Fig. 4. SM Figs. 5 and 6 markedly show that patients in the high-risk group had a poor prognosis, which demonstrated that this molecular model could rapidly identify severe patients.

### Pathway risk model

**Fig. 2:**
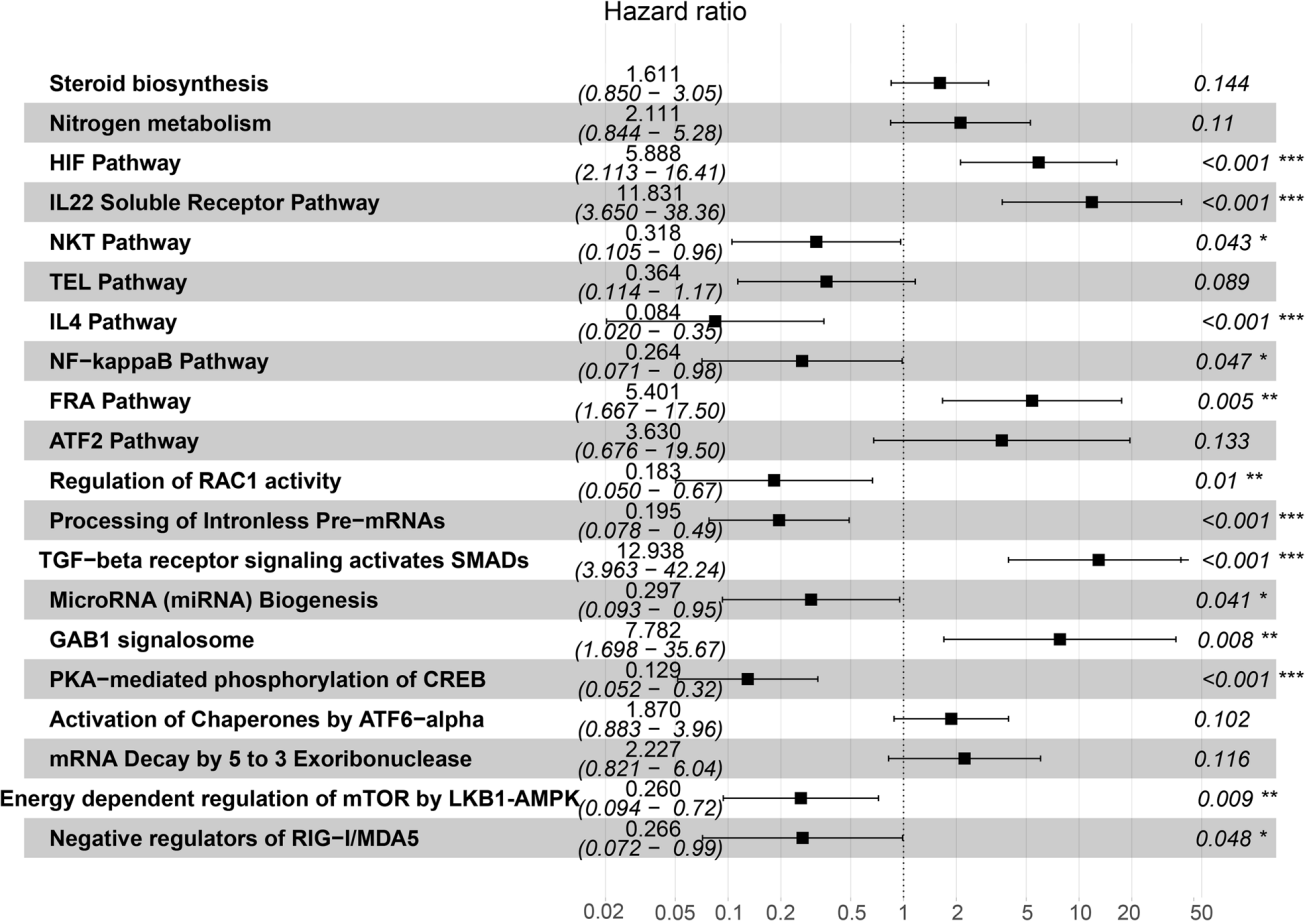
The Pathway-risk model. To construct the Pathway-risk model, two steps (Pathway characteristics screening and model establishing) were conducted. Pathway characteristics screening: 20 prognostic pathways were finally enrolled into the Pathway-risk model after univariate Cox regression analyses, Lasso regression analyses and both backward and forward stepwises selection based on the AIC. Pathway-risk model establishing: The final pathway model was constructed by multivariate Cox regression analyses and generated a pathway risk score for every patient. The forest plots of 20 pathways in multivariate Cox regression analyses. The first column was the name of 20 pathways; the second column showed the sample size; the third column illuminated the HR and 95%CI of 20 pathways; the forth column was the forest plots of HR and 95%CI; the last column indicated the *P* value of 20 pathways in multivariate Cox regression analyses

A total of 267 pathways that were identified through univariate Cox regression as prognostic pathways were significantly associated with 28-day cumulative mortality (*P* < 0.05), as shown in SM Table 3. Twenty pathways were further screened as final characteristics for the construction of the pathway model, as shown in Fig. 2, SM Fig. 7 and SM Table 4. The nomograms of the pathway model are shown in SM Fig. 8.

**Fig. 3:**
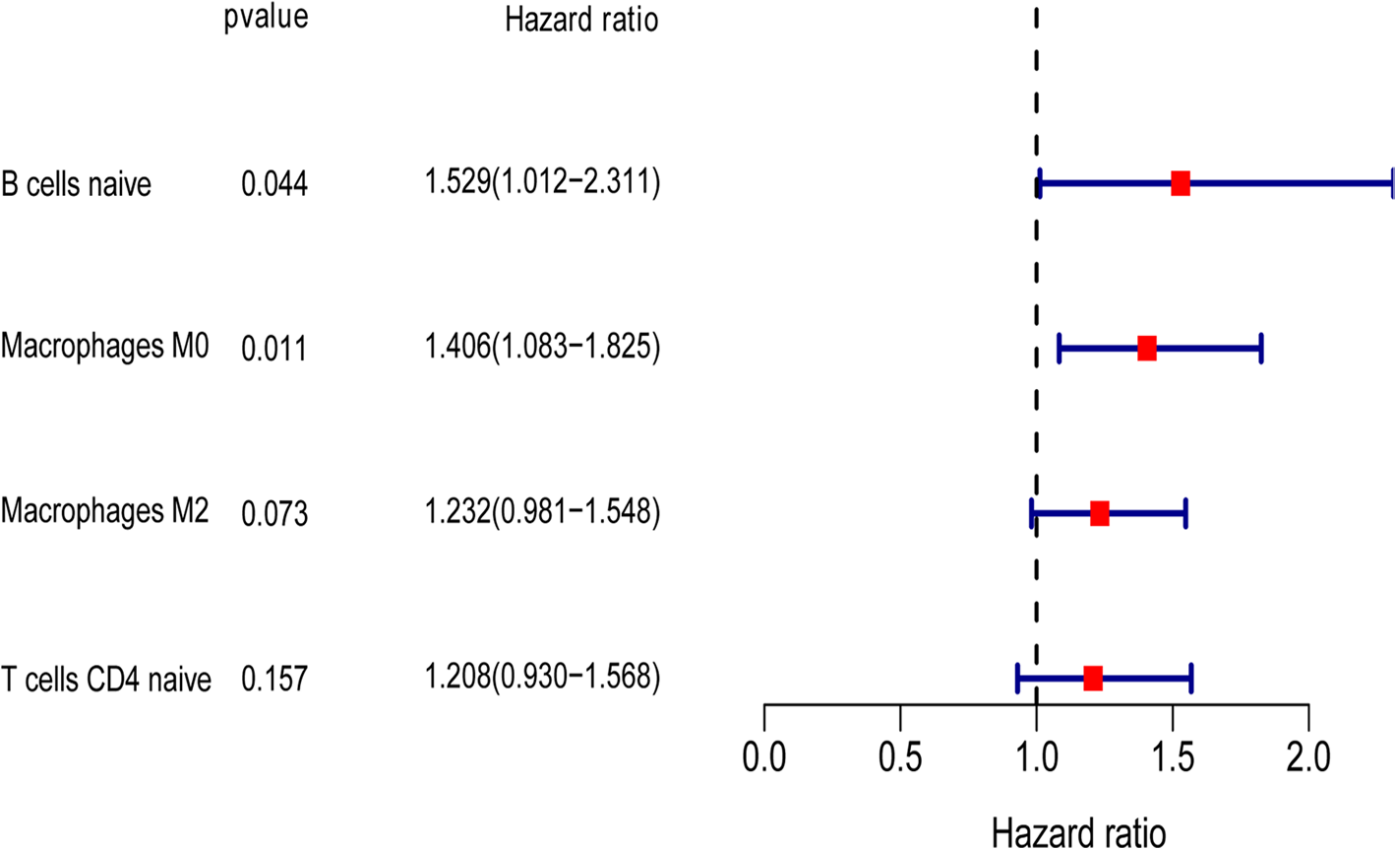
Network diagram to explain the upstream and downstream relationship of the Pathway-risk model. The red pathways meant hazarded pathways (HR > 1) and the blue pathways meant protective pathways (HR < 1). The pathways included in this model can be classified as two types, hypoxia and immune dysregulation. The pathway related to hypoxia was HIF-1 pathway. 1. Hypoxia further induced cellular injury and metabolic disorder. The pathways related to metabolic disorder included steroid biosynthesis pathway, Nitrogen metabolism pathway and energy dependent of regulation of mTOR by LKB1-AMPK. The pathways related to cellular injury included TEL pathway and ATF2 pathway. The cell repair was the negative feedback of cellular injury, and the pathways related to cellular injury included GAB1 signalosome and TGF-beta receptor signaling activates SMADs. 2. Immune dysregulation can be classified as anti-immunity, promoting-immunity and bipolar immune regulation. The pathways related to anti-immunity were TGF-beta receptor signaling activates SMADs pathway and IL22 soluble receptor pathway; the pathways related to promoting-immunity were NKT pathway, NF-kappaB pathway and PKA-mediated phosphorylation of CREB; the pathways related to bipolar immune regulation were IL-4 pathway and negative regulation of RIG-I/MDA5 pathway

SM Figs. 9 and 10 clearly show that the number of patients who died increased with increasing pathway risk, which demonstrated that the pathway model was highly associated with the outcome of septic patients. These pathways were associated with hypoxia and immune dysregulation in sepsis patients. The network relationship of the pathways is shown in Fig. 3.

### Immunity risk model

**Fig. 4:**
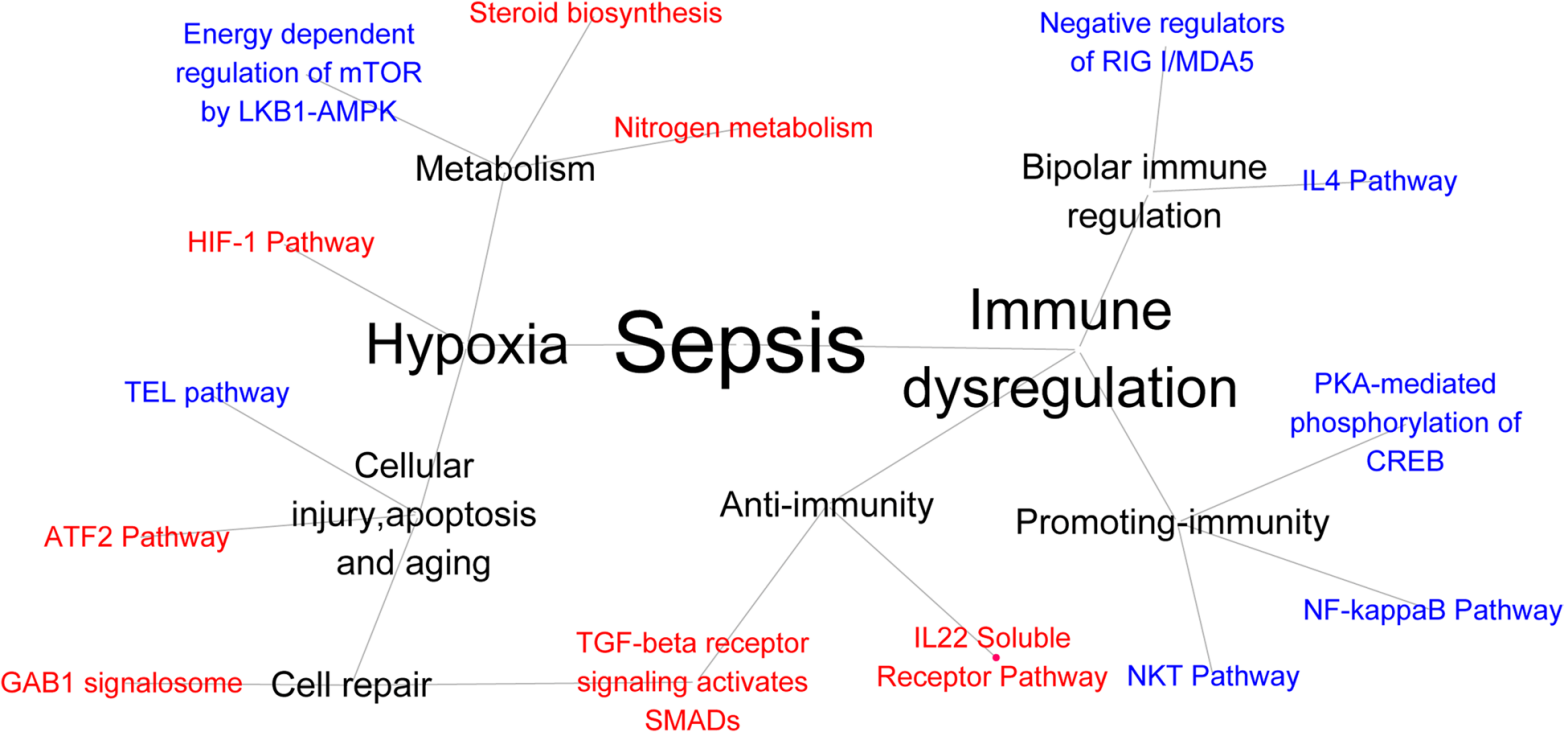
The Immunity-risk model. To construct the Immunity-risk model, two steps (prognostic immune cells screening and model establishing) were conducted. Prognostic immune cells screening: 4 prognostic immune cells were finally enrolled into the Immunity-risk model after univariate Cox regression analyses, Lasso regression analyses and both backward and forward stepwises selection based on the AIC. Immunity-risk model establishing: The final immune model was constructed by multivariate Cox regression analyses and generated a immunity risk score for every patient. The forest plots of 22 immune cells in univariate Cox regression analyses. The first column was the name of 22 immune cells; the second column showed the *P* value; the third column illuminated the HR and 95%CI of 20 pathways; the forth column was the forest plots of HR and 95%CI

Four immune cells (naïve CD4 T cells, naïve naïve B cells, M0 macrophages and M2 macrophages) were found to be significantly associated with 28-day cumulative mortality, as shown in Fig. 4. The nomograms of the immunity model are shown in SM Fig. 11. SM Figs. 12 and 13 clearly show that naïve CD4 T cells, naïve naïve B cells, and M0 and M2 macrophages were obviously upregulated in patients in the high-risk group.

### Combined risk model

To evaluate sepsis in multiple dimensions, we merged the risk factors from the molecular risk model, pathway risk model, immunity risk model and basic patient information (age, sex and pneumonia diagnoses) into the combined model. The combined risk model and nomograms were eventually built based on age, 8 prognostic molecules and 9 crucial pathways since these variables were independent prognostic factors for sepsis, as shown in SM Fig. 14.

**Fig. 5:**
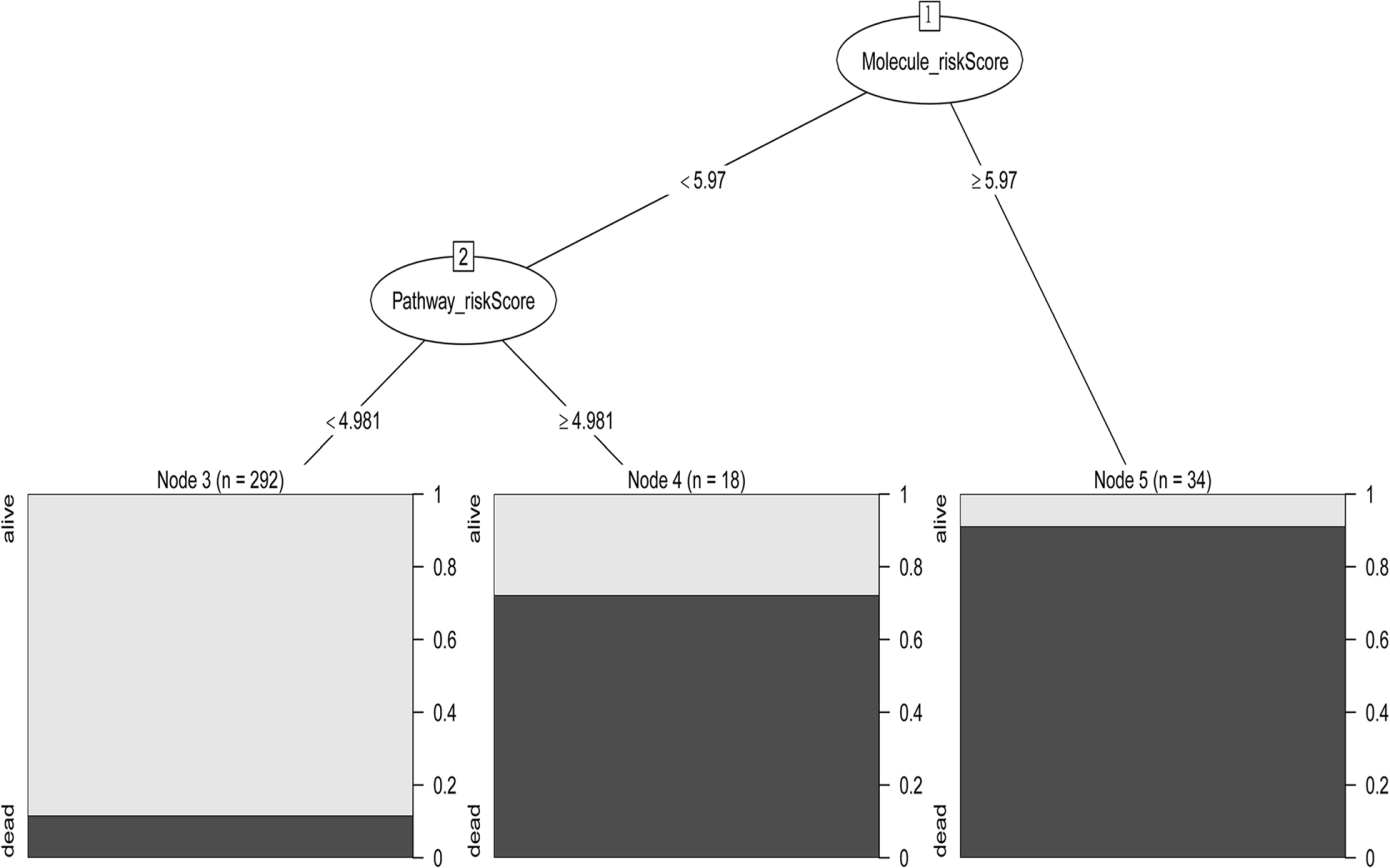
Decision Tree of Combined-risk model for rapid clinical application. To evaluate sepsis in multi-dimension, we merged the risk factors from molecule-risk model, pathway-risk model, immunity-risk model and basic information of patients (age, gender and pneumonia diagnoses) into the combined model. Based on Decision Tree, patients could be classified as 3 subgroups with markedly different outcomes, which could rapidly predict the outcome for septic patient

The decision tree algorithm was utilized to further simplify and optimize the combined risk model for rapid clinical application, as shown in Fig. 5. Based on the decision tree algorithm, patients could be classified into 3 subgroups with markedly different outcomes, which could rapidly predict the outcome for septic patients.

In addition, the therapeutic recommendations for patients with high risk scores calculated by the molecular risk model, pathway risk model or immunity risk model are summarized in Table 2.

**Table 2:** The therapeutic recommendations for patients with high risk score

Models	Therapeutic measures for patients with high risk score	Number of features
Molecule-risk	Be diverted to intensive care unit for maximal intervention	8
Pathway-risk	1.To improve oxygen delivery and decrease oxygen consumption; 2. More hemodynamic measurement to guide therapy.	9
Immunity-risk	1. To promote immunity therapy such as interferon and thymosin; 2. To be on guard against superinfection occurring.	4

In Table 2, we provided the therapeutic recommendations for patients with high risk score identified by three sub-models. In addition, the numbers of features in each sub-model were also shown in Table 2

### Assessment of model performance

**Fig. 6:**
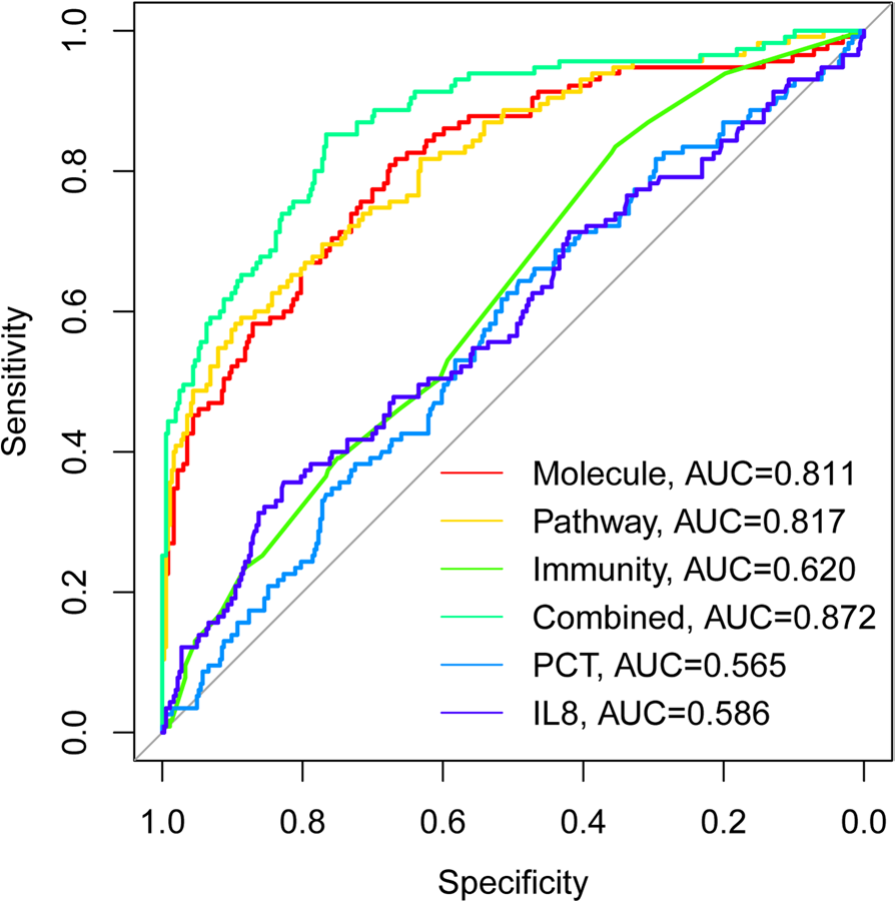
Model evaluation on 3 sub-models, Combined-risk model and traditional indictors (PCT and IL8) in external validation cohort. The ROC indicated that the AUC of Molecule-risk model, Pathway-risk model, Immunity-risk model, Combined-risk model, IL8 and PCT were 0.810, 0.818, 0.621, 0.873, 0.585 and 0.565

The C statistics of the molecular risk model, pathway risk model, immunity risk model and combined risk model were 0.79, 0.79, 0.61 and 0.873, respectively. The AUCs were 0.81, 0.82, 0.62 and 0.873, respectively (Fig. 6).

In addition, the current models were further compared in clinical value with traditional indicators such as procalcitonin (PCT) and interleukin-8 (IL-8). The AUCs of PCT and IL-8 were 0.565 and 0.585, respectively, which were markedly lower than those of the combined model and submodels (Fig. 6).

The above results indicated that the models in this study are all superior to traditional biomarkers for predicting the prognosis of sepsis patients.

## Discussion

Sepsis identifies a highly heterogeneous population of patients, including a wide possible extent of patient conditions, complications, severity levels, pathogens, and underlying immune states [4, 5]. To evaluate heterogeneity of sepsis in multi-dimension, the current study constructed a Combined-risk model including three sub-models (The molecule-risk model, the Pathway-risk model and the Immunity-risk model). The molecule-risk score could provide an opportunity to estimate prognosis rapidly for precise evaluation the utility of aggressive interventions. The Pathway-risk model could adequately quantify the patient’s underlying pathway activity alterations for matched therapies selection. The immunity risk score from current nomograms could predict immune suppressed states based on immune cell subsets. This model could be used as the pre-hospital screening tool to identify sepsis heterogeneity, and also could be utilized as the bedsides monitoring tool to continuously evaluate sepsis. The further investigation will develop the related kits for rapid detection the screened molecules, pathways and immune cells.

For rapidly estimate prognosis of septic patients, the previous prediction models and classical biomarkers could not precisely identify severe patients in external dataset [32–36]. The current Molecule-risk model performed well in one external cohort with C-index and AUC as 0.790 and 0.810. To furthermore validate the novel value of the current model, the current study compared our models with the traditional indicators including procalcitonin (PCT) and interleukin-8 (IL-8) in discriminative ability via receiver operating characteristic curves, shown in Fig. 6. The results indicated that the model performances of the Molecule-risk model and other models were obviously superior to the traditional indicators in clinical value. Since the patients with high risk scores calculated by molecular model were very sick sepsis, the recommended treatments for could be transferred to ICU for more intensive monitor and treatments, while patients predicted to own favorable prognosis were watched in the general wards safely.

For identifying the heterogeneous pathogenesis in sepsis, previous studies [9, 35–37] for risk stratification including clinical severity scores such as Acute Physiology and Chronic Health Evaluation (APACHE) or Sequential Organ Failure Assessment (SOFA) as well as molecular biomarkers could not categorize sepsis at pathogenesis level. It is well known that even in patients with similar clinical features, underlying pathophysiological mechanisms such as signal transduction pathway are significant different. The current pathway-risk model intrinsically uncovered a part of heterogeneous pathogenesis for further selection of specific therapy. The Pathway-risk score showed that signal transduction pathways of hypoxia, cellular injury and negative feedback repair, metabolic disorder and immune dysregulation were significantly associated with adverse outcomes of sepsis (Fig. 3). These data demonstrate several pathways in sepsis consistent with current knowledge of the pathology of this disease [38–45]. In addition, pathway-risk score uncovered biological pathway activity variation for accurate identification of high risk patients at pathogenesis level. Compared with surface appearances such as lactic acid, hypoxia inducible factor-1 pathway was classically recognized as intrinsic and robust indictor of tissue perfusion insufficiency. Similarly, steroid biosynthesis pathway, nitrogen metabolism pathway and liver kinase B1- AMP-activated protein kinase pathway could identify heterogeneity of metabolic disorder for sepsis. Simultaneously, mitochondrial membrane damage-related pathway (activating transcription factor-2 pathway), apoptosis-related pathway (telomeres, telomerase, cellular aging, and immortality pathway) and negative feedback repair-related pathway (GRB2 associated binding protein 1 signalosome and transforming growth factor-beta receptor signaling activates SMADs) could uncover some heterogeneity of cellular injury for sepsis. As patients with high risk scores calculated by pathway model could have hypoxia and lower organ perfusion, the current study recommended to provide more hemodynamic measurement, improve oxygen delivery and decrease oxygen consumption for these patients.

For classifying immune status in sepsis, conventional immunity-model [46, 47] built by inflammatory markers could not uncover immune cells dysfunction or/and dys-homeostasis, the immunity risk score from current nomograms could predict immune suppressed states based on immune cell subsets. Consistent with previous studies of immunity of sepsis, the Immunity-risk score demonstrated that states of prolonged immune dysfunction leading to immune paralysis are more detrimental to sepsis survival [15]. Immune paralysis is characterized that naïve immune cells (M0 macrophages, B cells naïve and T cells naïve) are hardly activated into mature inflammatory cells, accompanying with an absolutely increasing proportion of immunosuppressive cells such as M2 macrophages and Treg [15, 19, 36, 37]. Consequently, the patients with high Immunity-risk score should be alert of superinfection due to states of immune suppression. Subsequently, promoted immunity such as interferon or thymosin and avoidance of probable infection such as removing inessential catheters could be considered possibly. On the contrary, as a therapy for improving cardiovascular responses to diverse stress stimulation, corticosteroids could be safely used for patients with low Immunity-risk score.

In addition, for rapid clinical application, Decision Tree algorithm of Combined-risk model was utilized to furthermore simplify the model, which could assist clinicians conveniently and efficiently [29].

The value of GSVA algorithm is a classical non-parametric and unsupervised functional enrichment analysis [17, 18]. Because GSVA could not depend on interest gene subset such as differential genes but on the whole ranking of genes, GSVA has been demonstrated to provide better sensitivity to screen gene expression variations of tiny magnitude which perform coordinately in signal transduction pathway related genes [40, 41]. Conventional ways to investigate immune cell disorders, such as immunohistochemistry and flow cytometry, depend on limited phenotypic markers, and sample disaggregation prior to experiments result in lost or damaged cells, which could change the conclusions of flow cytometry. CIBERSORT outperformed other ways for cell type identification of tissue and blood due to this computational approach reference to gene expression profiles of specific cell rather than one or two markers [19–21].

Overfitting problems unavoidably encountered in prediction models, such as inflation of regression coefficients with while deflation of standard errors, which ultimately reducing both the parsimony of the model and the generalizability of conclusions. Lasso algorithm was more effective for these problems especially in high-dimensional data such as gene expression profiles owing to stringency of lasso penalties. In addition, both backward and forward stepwises selection with AIC could further reduced authentic predictor variables into the final model, and simultaneously preserved model performance.

## Conclusions

As a secondary analysis of publicly available data, detailed information such as the severity, complications, and individual treatment of each patient could not be downloaded for further analysis. In addition, despite the present combined risk model performing well on one external validation, progressive research should be conducted in the future.

## Supplementary Information

**Additional file 1.**

**Additional file 2.**

**Additional file 3.**

## Data Availability

The original data were downloaded from the public database-GEO database (https://www.ncbi.nlm.nih.gov/geo/). The GSE number was GSE63042 and GSE65682.

## Abbreviations

GEO: Gene Expression Omnibus
AUC: The area under the receiver operating characteristic curve
PCT: Procalcitonin
IL-8: Interleukin-8
SM: Supplemental Material
GSVA: Gene set variation analysis
CIBERSORT: Cell type identification by estimating relative subset of known RNA transcripts
IQR: Low interquartile ranges
FDR: False discovery rate
LASSO: Least absolute shrinkage and selection operator
AIC: Akaike information criterion
HR: Hazard ratio
APACHE: Acute Physiology and Chronic Health Evaluation
SOFA: Sequential Organ Failure Assessment
