# Supplementary material for: A combined risk model for the multi-encompassing identification of heterogeneities of prognoses, biological pathway variations and immune states for sepsis patients
Source: BMC Anesthesiol. 2022 Jan 7;22:16. doi: 10.1186/s12871-021-01552-x (PMC8739717; doi:10.1186/s12871-021-01552-x)
Supplement: Supplementary file 1 — Additional file 1. [file 12871_2021_1552_MOESM1_ESM.doc]

The catalogue of Supplemental Material

| **Title** | **Content** | **Page** |
| --- | --- | --- |
| **SM Figure 1** | The analyses flow plot | 2 |
| **SM Figure 2** | The difference analysis of molecule expression between sepsis and health participants | 4 |
| **SM Figure 3** | Lasso regressions and cross validation of candidate molecules | 5 |
| **SM Figure 4** | Nomograms of the Molecule-risk model | 6 |
| **SM Figure 5** | Classify patients into low and high risk groups through Molecule-risk model. | 7 |
| **SM Figure 6** | Expression of 15 molecules in low and high risk groups were shown in heat-map. | 8 |
| **SM Figure 7** | Lasso regressions and cross validation of candidate pathways | 9 |
| **SM Figure 8** | Nomograms of the Pathway-risk model | 10 |
| **SM Figure 9** | Classify patients into low to high risk group through Pathway-risk model | 11 |
| **SM Figure 10** | Relative expression of 20 pathways in low and high risk groups were shown in heat-map | 12 |
| **SM Figure 11** | Nomograms of the Immunity-risk model | 13 |
| **SM Figure 12** | Classify patients into low to high risk group through Immunity-risk model | 14 |
| **SM Figure 13** | Relative expression of immune cells in low and high risk groups were shown in heat-map | 15 |
| **SM Figure 14** | The combined-risk model | 16 |
| **SM Table 1** | Prognostic molecules in sepsis screened by univariate Cox regression analyses. | 17 |
| **SM Table 2** | Candidate molecules screened by Lasso regression analyses with cross validation. | 25 |
| **SM Table 3**  **SM Table 4** | Prognostic pathways in sepsis screened by univariate Cox regression  Pathways in sepsis screened by Lasso regression analyses with cross validation. | 31  42 |
| **SM content1** | The R code of this study | 44 |
| **SM content2** | Datasets description | 52 |

SM=Supplemental Material


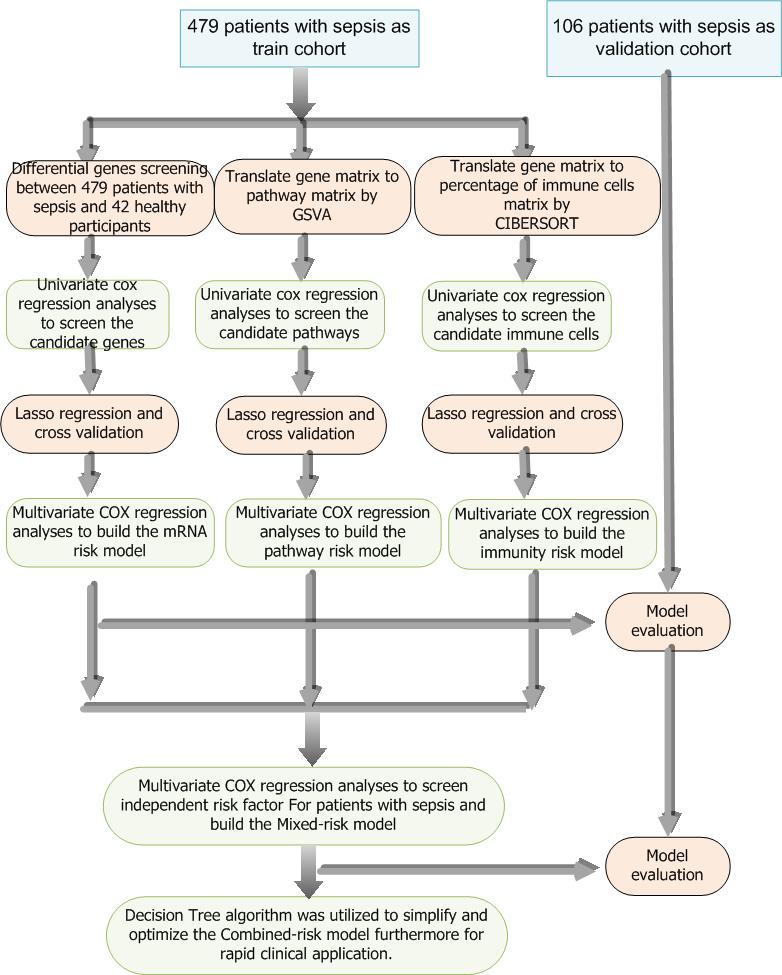


**SM Figure 1. The analyses flow plot**

The model construction included three steps: (1) Screen characteristics; (2) Build sub-models and combined model; (3) Simplify model

(1)Screen characteristics

Firstly, the differentially expressed mRNA was screened based on moderated t-tests, with false discovery rate (FDR) < 0.05 as significance criteria. GSVA and CIBERSORT algorithm were utilized to calculate the pathway scores and immune cell percents, respectively.

(2)Build sub-models and combined model

In train dataset, the associations of relevant characteristics with survival would be evaluated based on Cox proportional hazards regression models. Hazard ratios (HRs) were shown with their 95%CIs. Selected characteristics would be incorporated in the nomograms(risk model) to predict the probability of 28 day mortality using rms R package.

To evaluate sepsis in multi-dimension, we merged the risk factors from molecule-risk model, pathway-risk model, immunity-risk model and basic information of patients (age, gender and pneumonia diagnoses) into the combined model. The Combined-risk model and nomograms were eventually built based on ages, 8 prognostic molecules and 9 crucial pathways since these variables were independent prognostic factors for sepsis.

(3) Simplify model

Decision Tree algorithm was utilized to simplify and optimize the Combined-risk model furthermore for rapid clinical application.


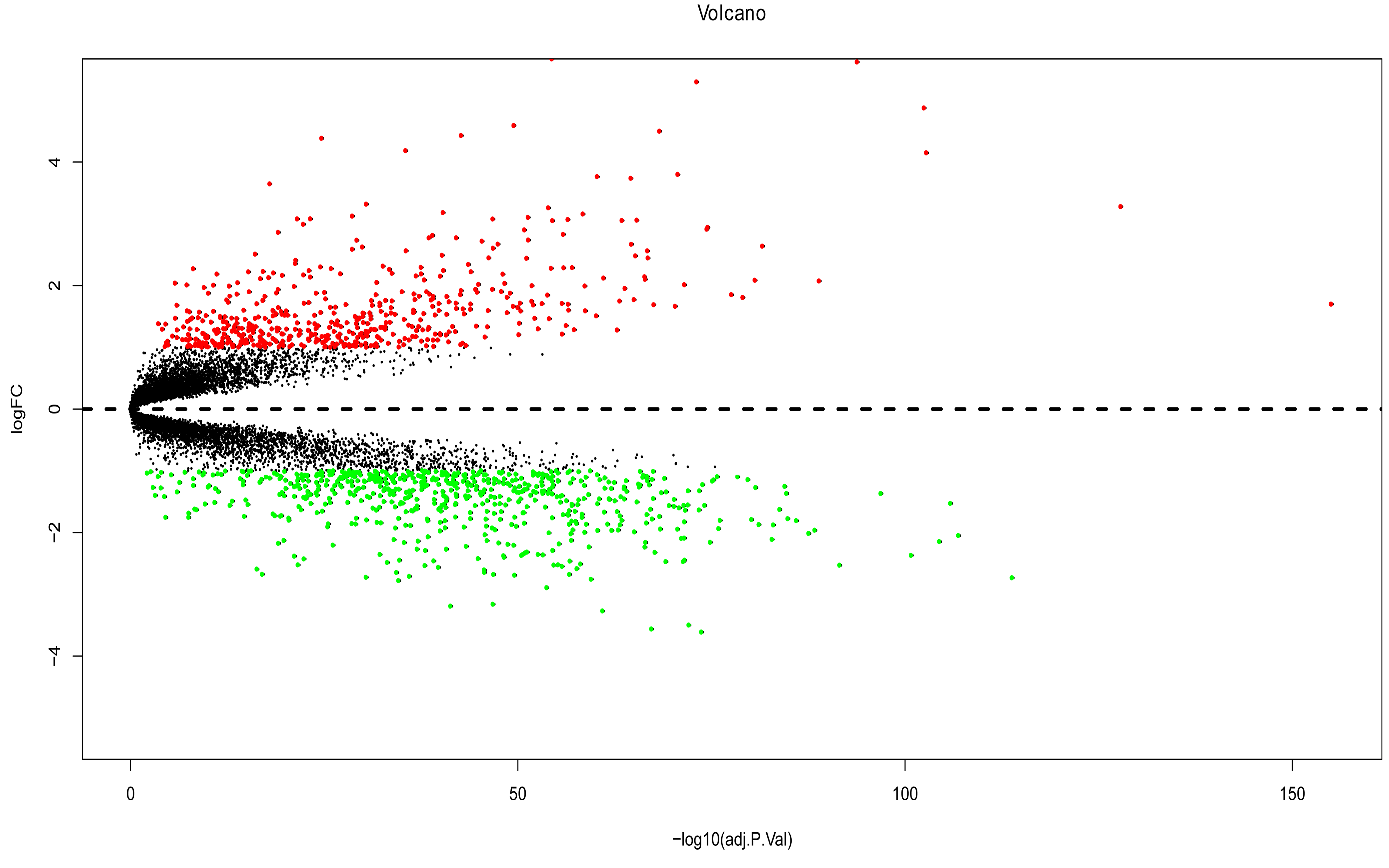


**SM Figure 2. The difference analysis of molecule expression between sepsis and health participants**

Red dots represented high expression molecules, and blue dots represented low expression molecules. The filters were log2 fold change (LFC) >2 and false discovery rate (FDR) < 0.05.


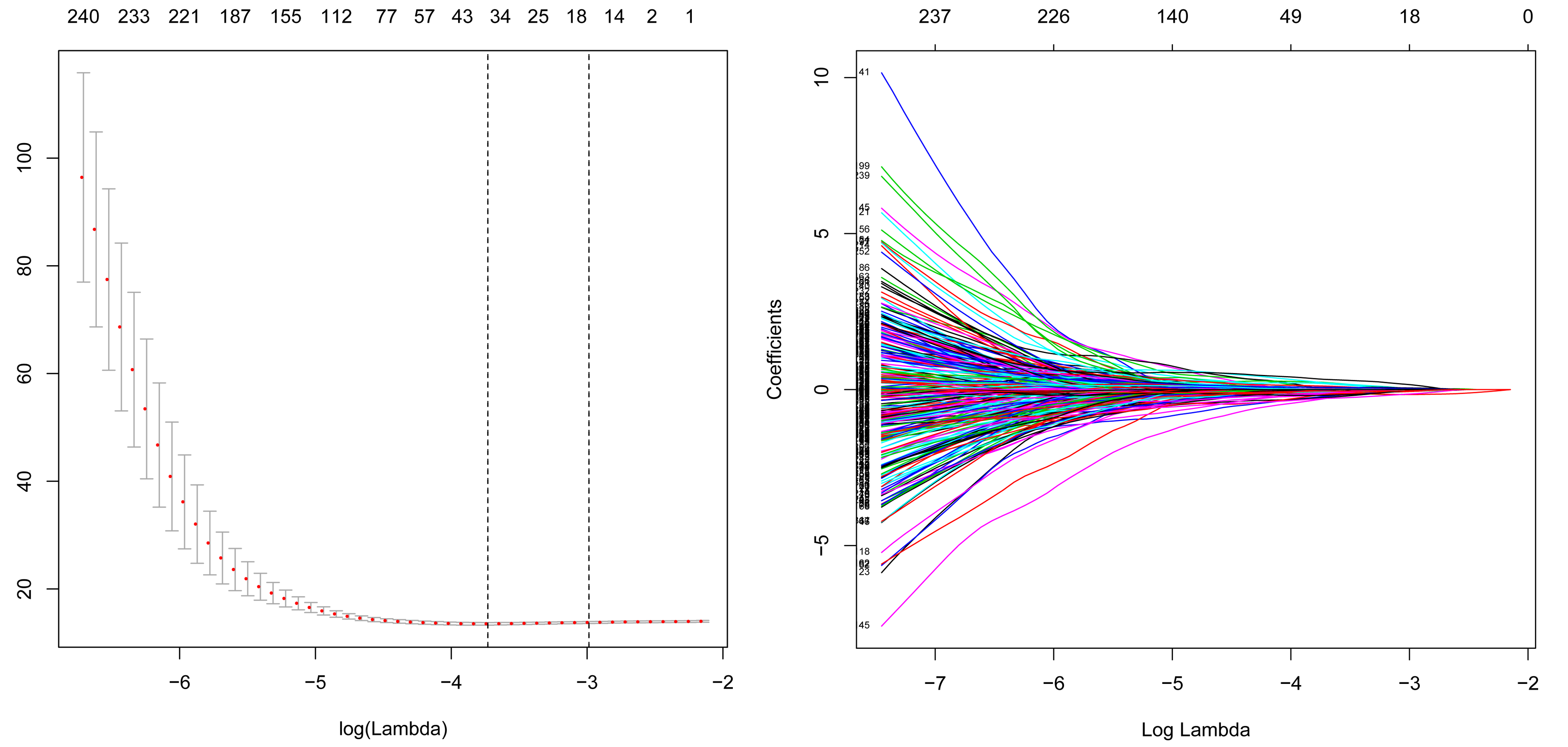


**SM Figure 3. Lasso regressions and cross validation of candidate molecules**

Lasso regression analyses and cross validation indicated that the model of 34 molecules had the minimum of partial likelihood deviance


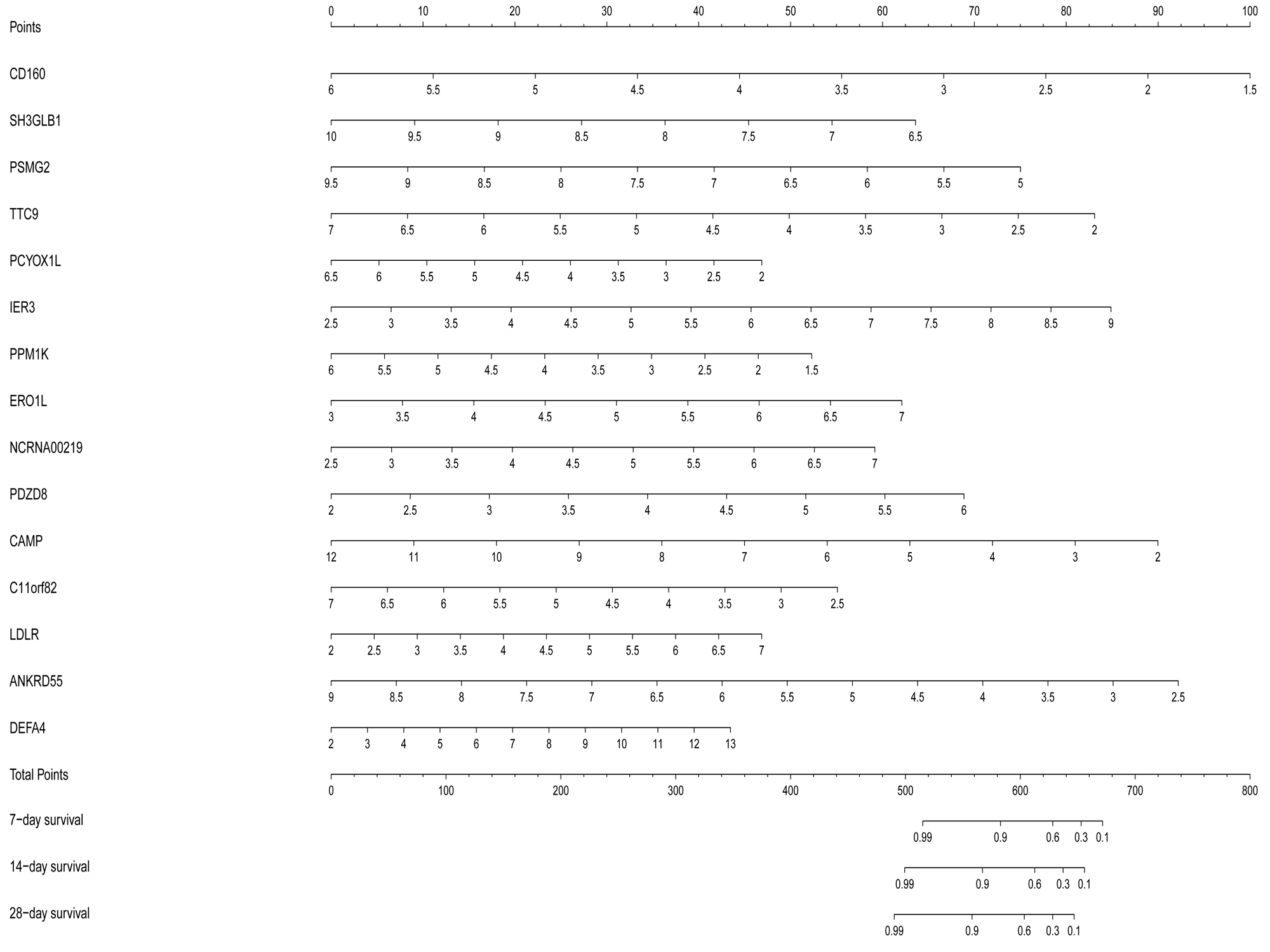


**SM Figure 4. Nomograms of the Molecule-risk model**


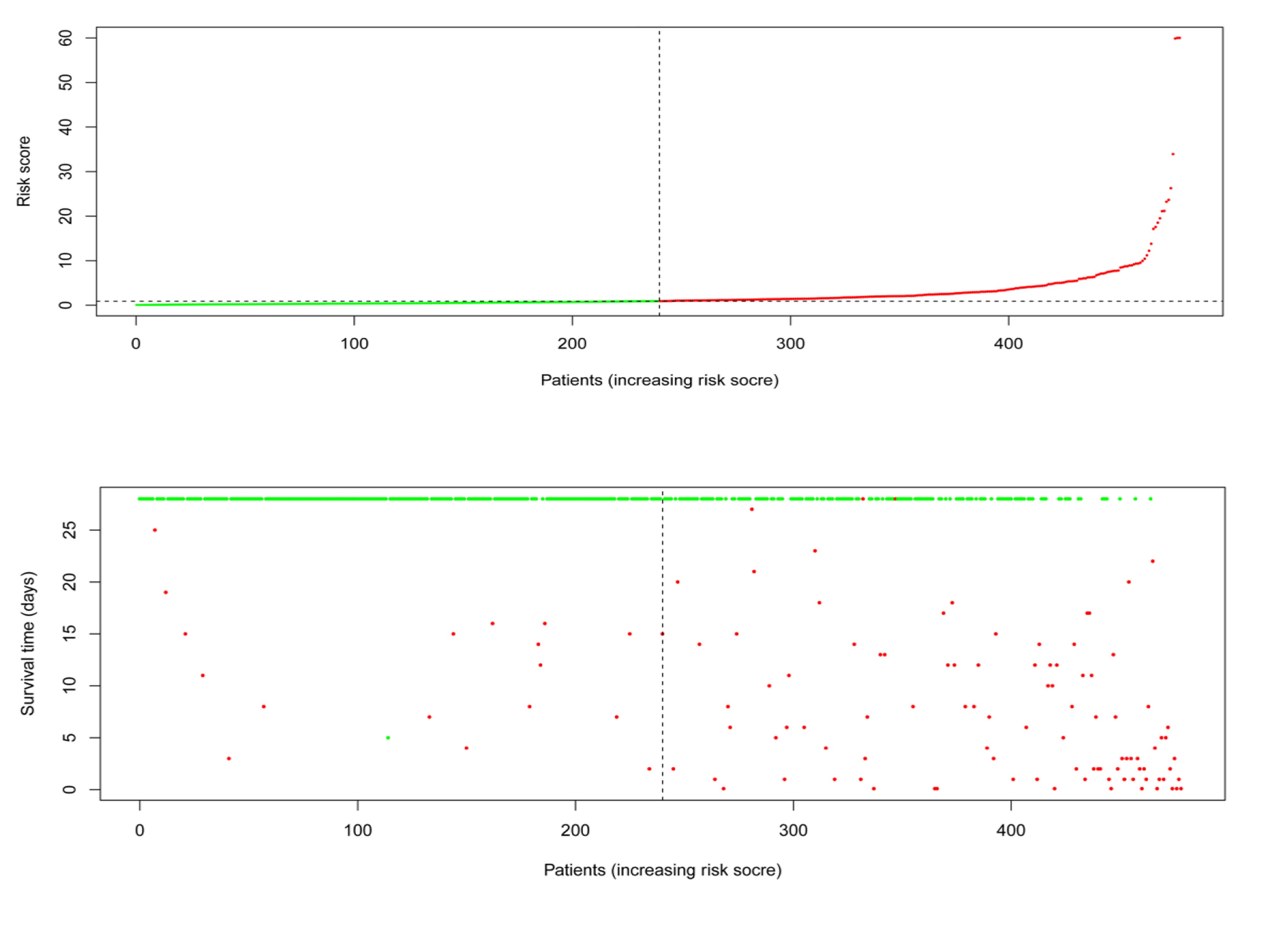


**SM Figure 5. Classify patients into low and high risk groups through Molecule-risk model.**

Based on the Molecule-risk model, R software calculated the risk score for every patient. According to these risk scores, patients were ordered by the risk scores from low to high. Furthermore, patients were divided into high risk group and low risk group through median of risk scores (Dotted line). The longitudinal coordinates showed the risk score of every patient calculated by the Molecule-risk model. The horizontal ordinate indicated the patients ordered from low score to high score. The risk score calculated by the current Molecule-risk model markedly identified the patients with poor prognosis. The longitudinal coordinates showed the risk score of every patient from low to high. The horizontal ordinate showed the survival time of every patient. Red dots represented dead patients and blue dots represented survivors. From the lowest risk score to highest risk score, the number of dead patients was significantly increasing, and the survival days were significantly decreasing.


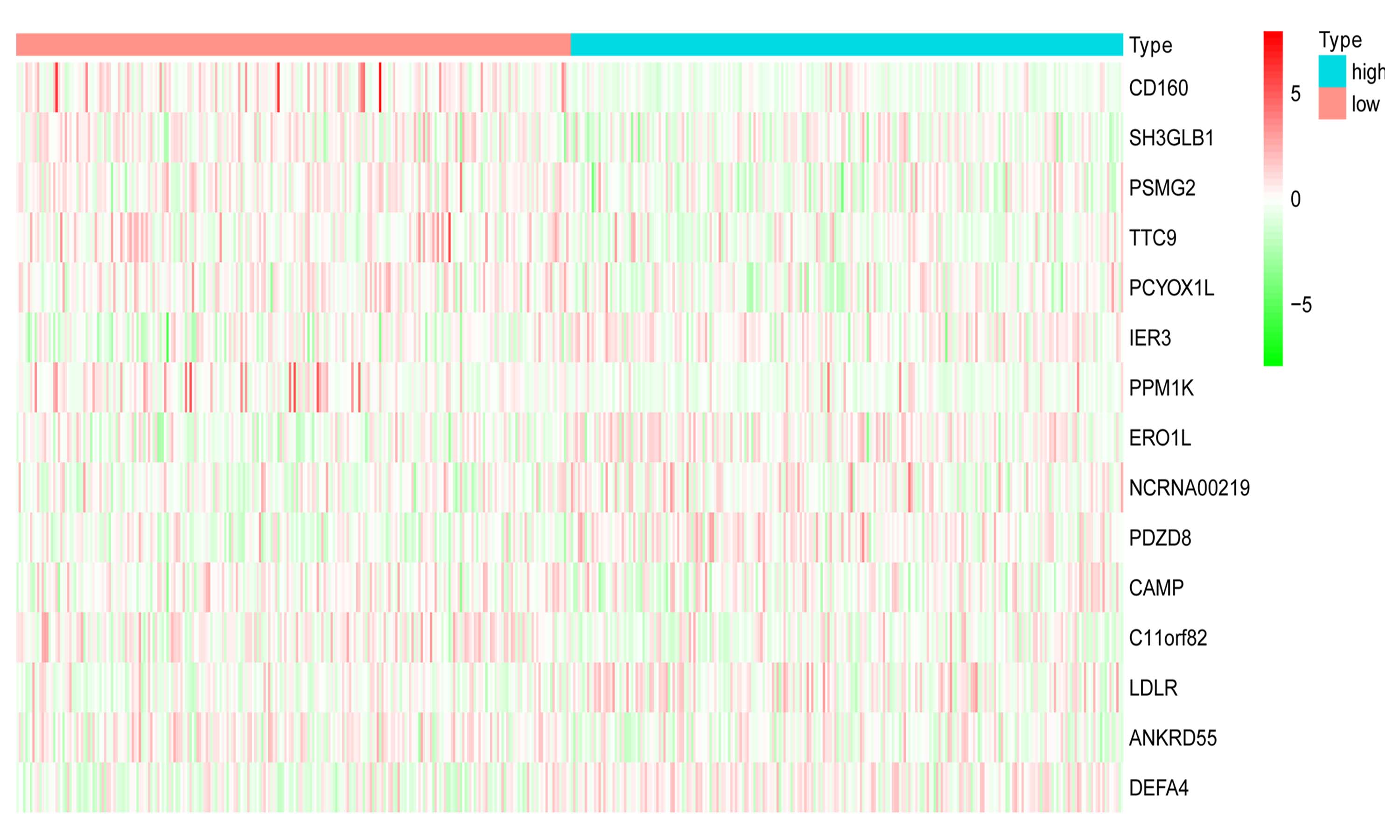


**SM Figure 6. Expression of 15 molecules in low and high risk groups were shown in heat-map.**


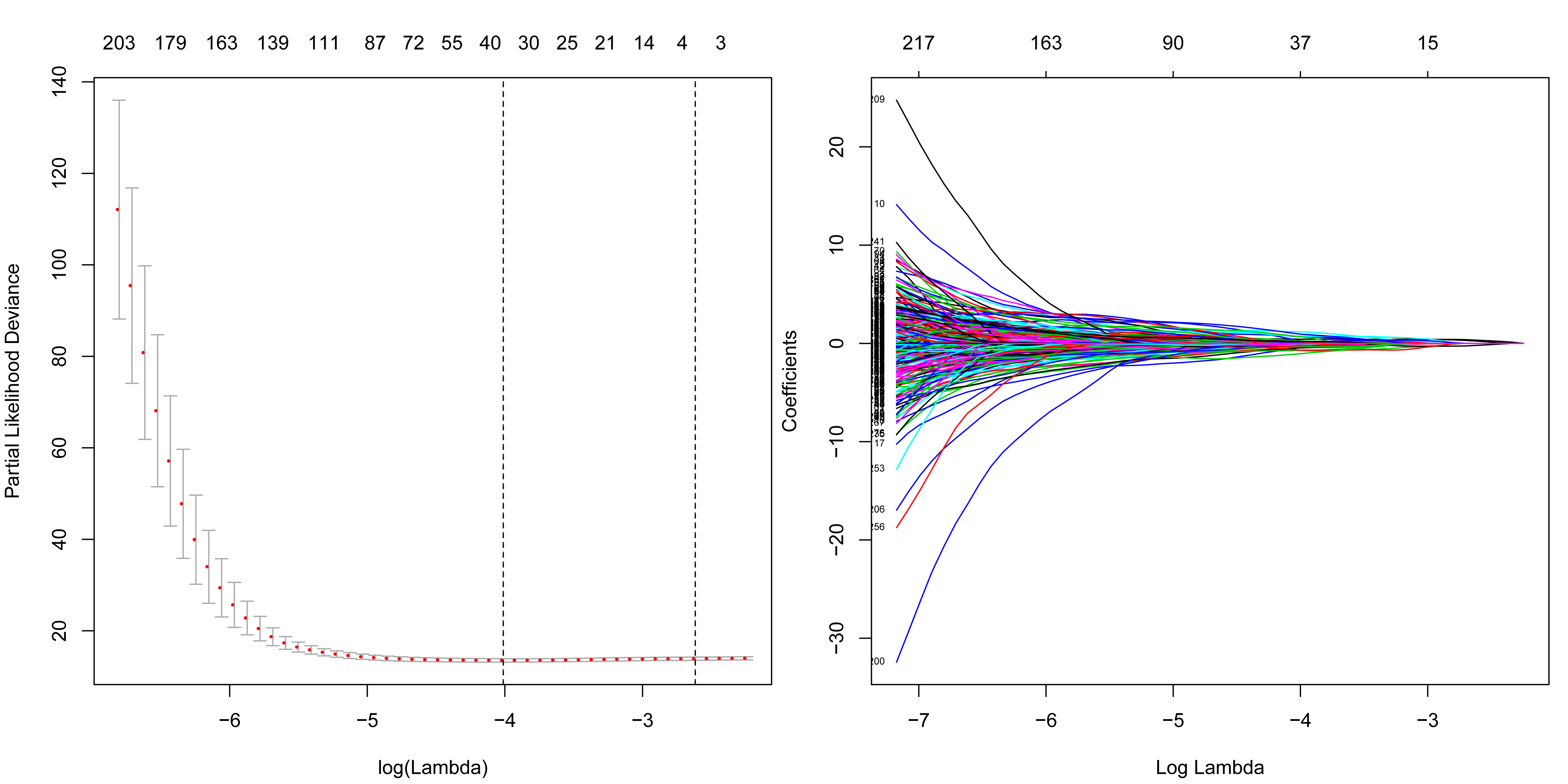


**SM Figure 7. Lasso regressions and cross validation of candidate pathways**

Lasso regression analyses and cross validation indicated that the model of 37 pathways had the minimum of partial likelihood deviance


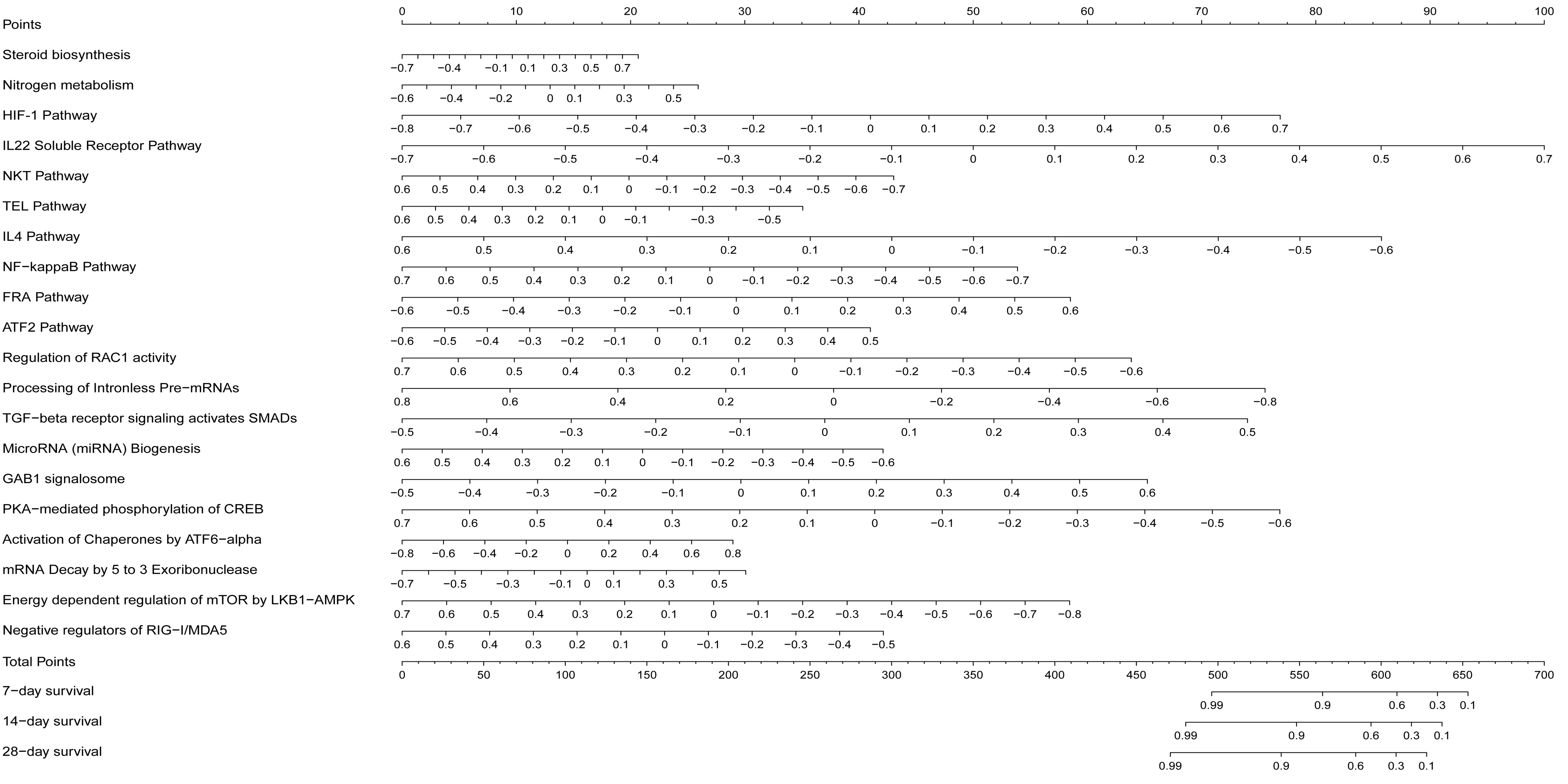


**SM Figure 8. Nomograms of the Pathway-risk model**


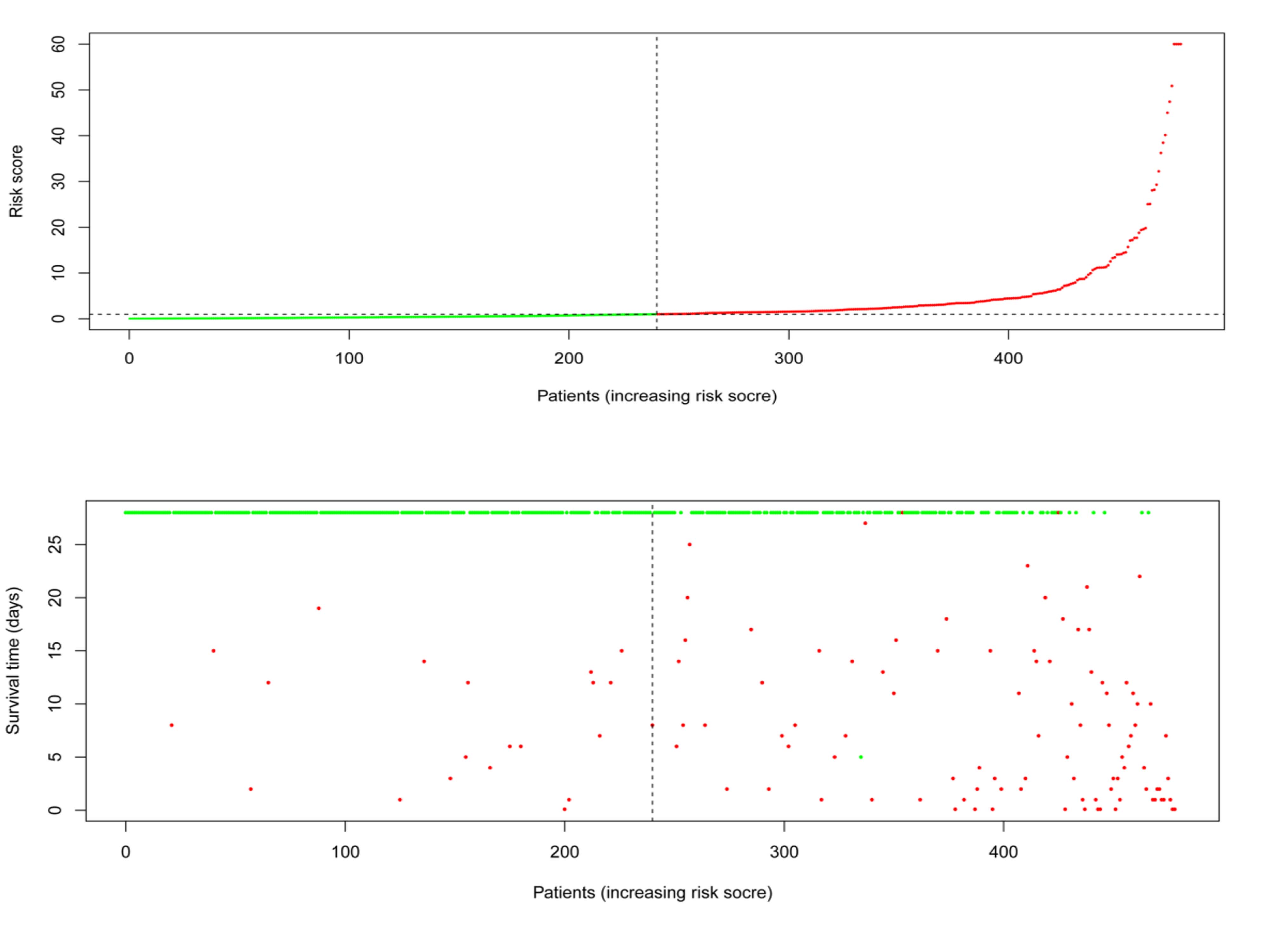


**SM Figure 9. Classify patients into low to high risk group through Pathway-risk model.**

Based on the Pathway-risk model, R software calculated the risk score for every patient. According to these risk scores, patients were ordered by the risk scores from low to high. Furthermore, patients were divided into high risk group and low risk group through median of risk scores (Dotted line). The longitudinal coordinates showed the risk score of every patient calculated by the Pathway-risk model. The horizontal ordinate indicated the patients ordered from low score to high score. The risk score calculated by the current Pathway-risk model markedly identified the patients with poor prognosis. The longitudinal coordinates showed the risk score of every patient from low to high. The horizontal ordinate showed the survival time of every patient. Red dots represented dead patients and blue dots represented survivors. From the lowest risk score to highest risk score, the number of dead patients was significantly increasing, and the survival days were obviously decreasing.

**
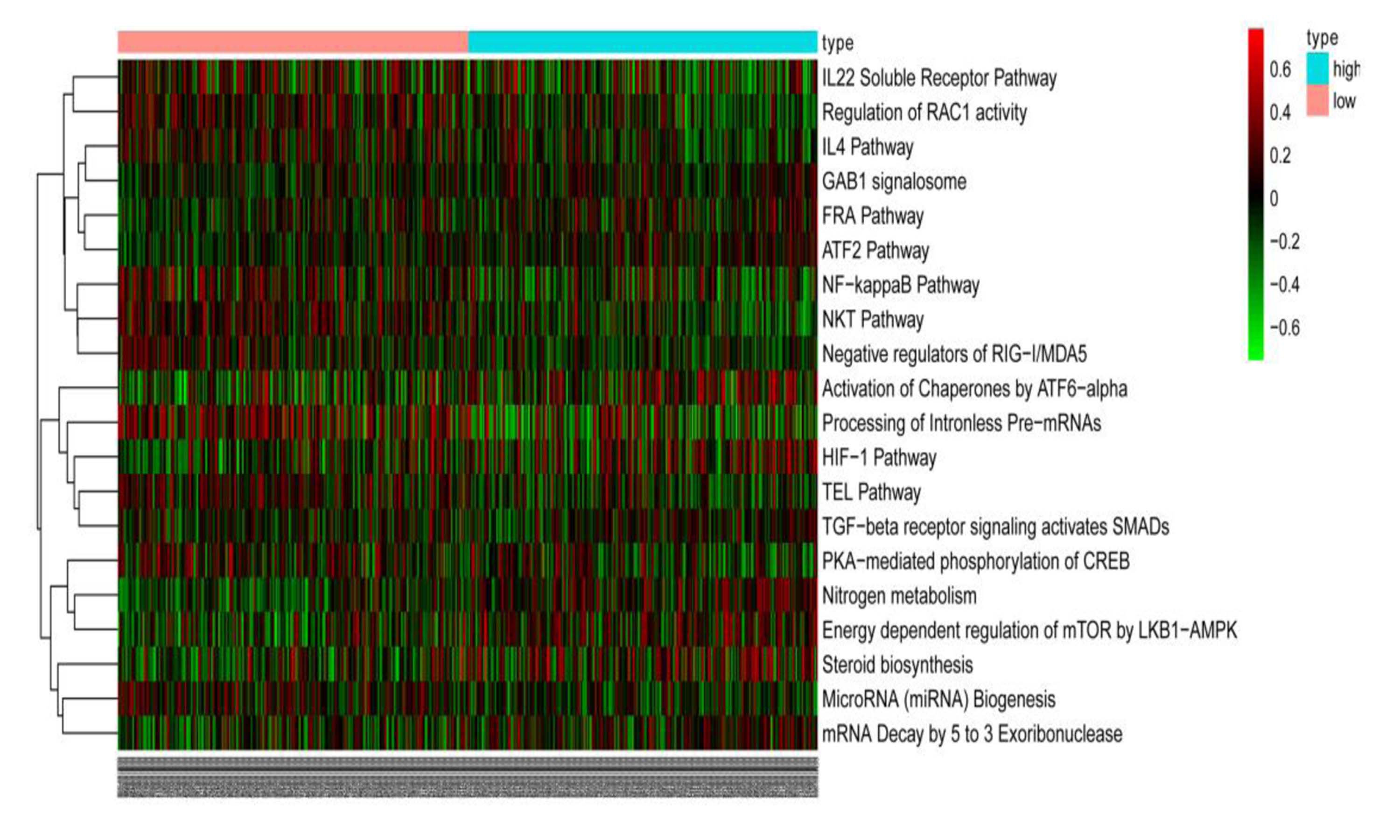
**

**SM Figure 10. Relative expression of 20 pathways in low and high risk groups were shown in heat-map.**


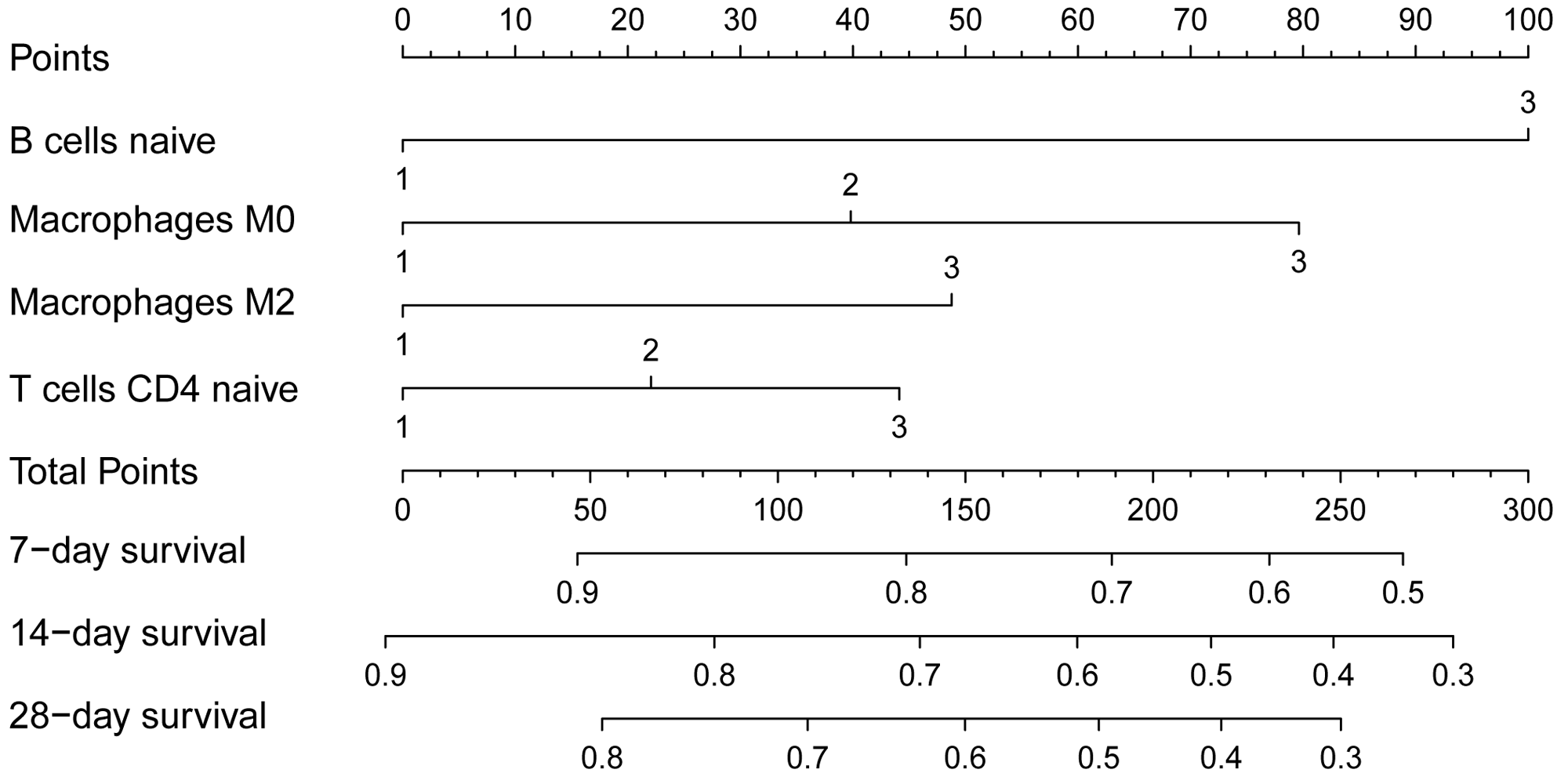


**SM Figure 11. Nomograms of the Immunity-risk model**

**
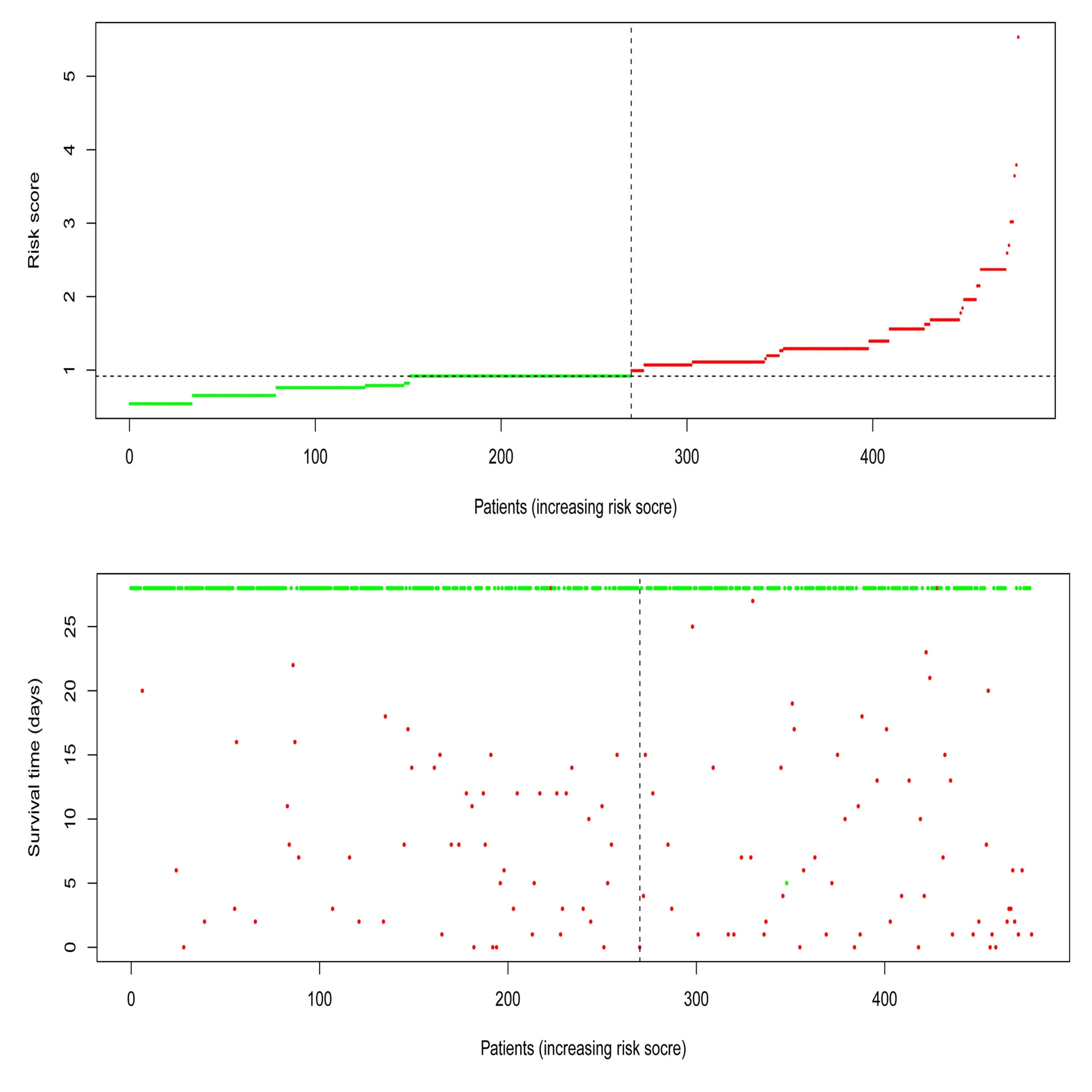
**

**SM Figure 12. Classify patients into low to high risk group through Immunity-risk model.**


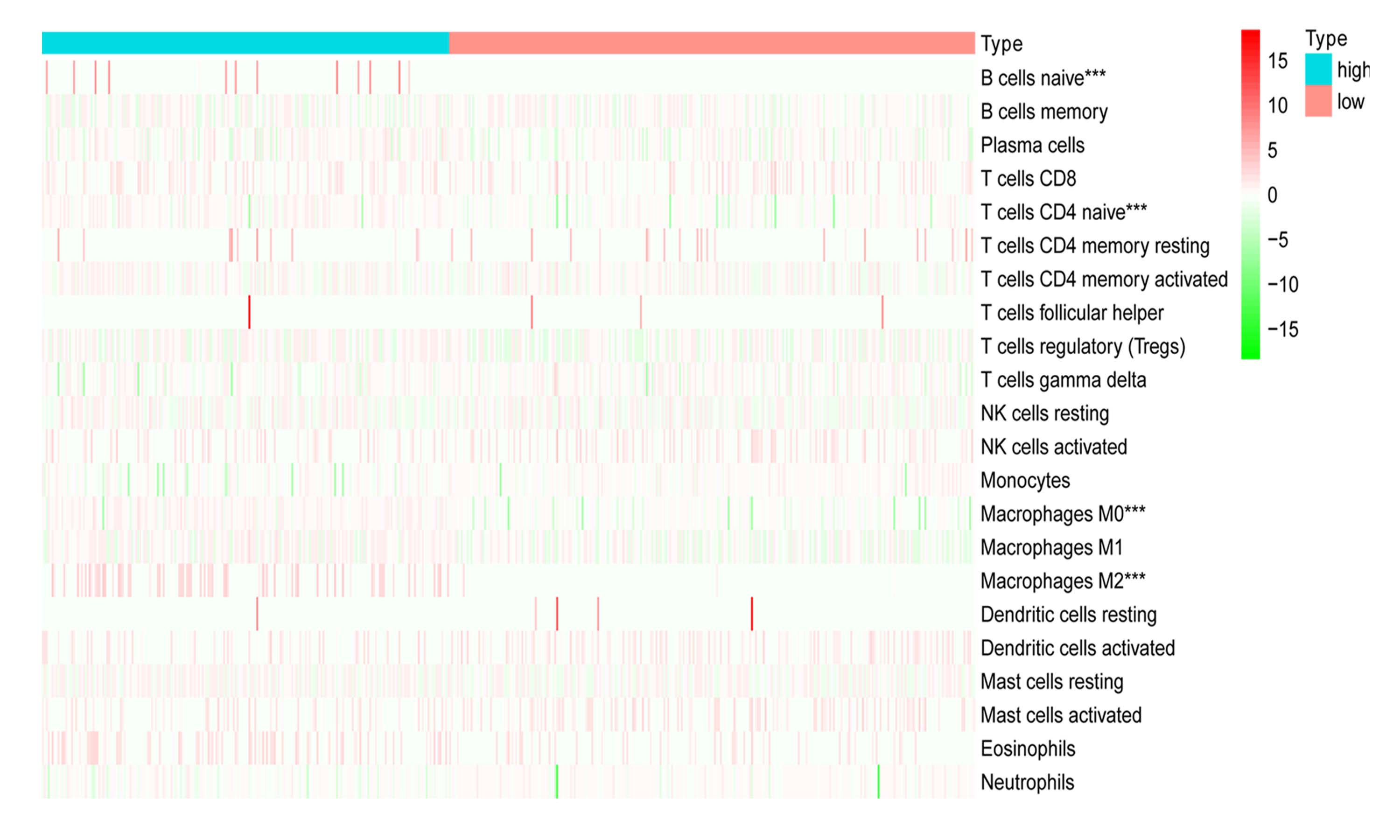


**SM Figure 13. Relative expression of immune cells in low and high risk groups were shown in heat-map.**


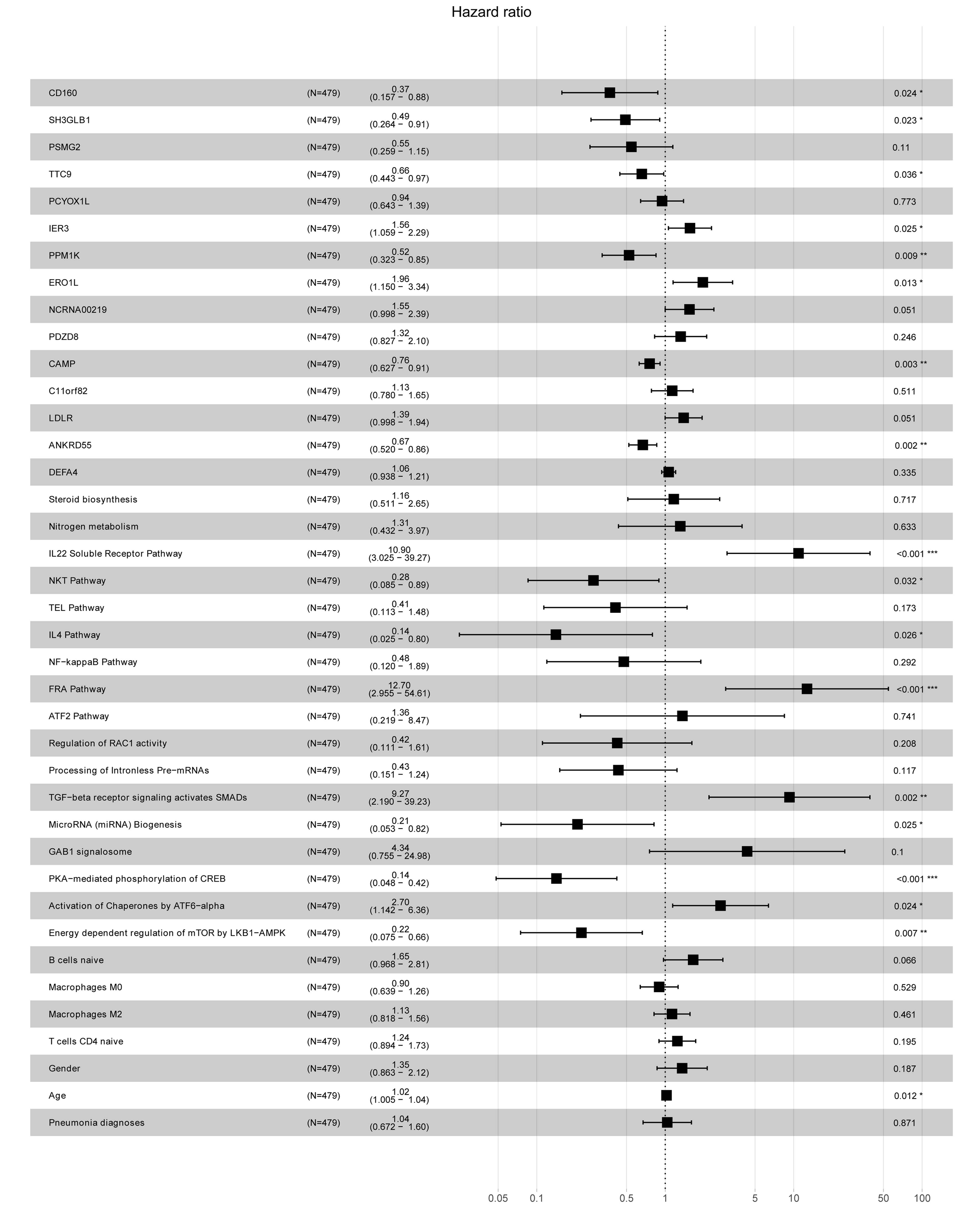


**SM Figure 14. The combined-risk model**

**SM Table 1. Prognostic molecules in sepsis screened by univariate Cox regression analyses.**

| **Genes screened by univariate Cox regression** | **HR** | **z** | **pvalue** |
| --- | --- | --- | --- |
| **CX3CR1** | 0.138041 | -5.70342 | 1.17E-08 |
| **PILRA** | 0.193782 | -4.57248 | 4.82E-06 |
| **GIMAP4** | 0.145796 | -4.41627 | 1.00E-05 |
| **CPPED1** | 0.058768 | -4.39519 | 1.11E-05 |
| **RABL2B** | 0.042638 | -4.33372 | 1.47E-05 |
| **ASTE1** | 0.10802 | -4.27946 | 1.87E-05 |
| **DENND2D** | 0.035527 | -4.26903 | 1.96E-05 |
| **AKNA** | 0.056883 | -4.2498 | 2.14E-05 |
| **MAOA** | 2.771057 | 4.106373 | 4.02E-05 |
| **ARL4A** | 4.86548 | 4.076121 | 4.58E-05 |
| **LTB** | 0.050986 | -4.04161 | 5.31E-05 |
| **CALHM2** | 0.139303 | -4.02168 | 5.78E-05 |
| **TMEM56** | 4.924053 | 3.988922 | 6.64E-05 |
| **GIMAP1** | 0.095152 | -3.96511 | 7.34E-05 |
| **MAFG** | 66.10435 | 3.961811 | 7.44E-05 |
| **ELANE** | 2.312689 | 3.919716 | 8.87E-05 |
| **UBE2C** | 6.268291 | 3.854074 | 0.000116 |
| **CD160** | 0.040604 | -3.84021 | 0.000123 |
| **KRT23** | 0.420045 | -3.83676 | 0.000125 |
| **CRTAM** | 0.081054 | -3.83208 | 0.000127 |
| **HIST1H3D** | 0.182263 | -3.82972 | 0.000128 |
| **RAB13** | 6.408465 | 3.810205 | 0.000139 |
| **CEACAM6** | 2.878678 | 3.792945 | 0.000149 |
| **NME4** | 3.871853 | 3.772231 | 0.000162 |
| **MS4A3** | 2.077886 | 3.760176 | 0.00017 |
| **CTSG** | 2.096477 | 3.742577 | 0.000182 |
| **BTN3A3** | 0.19061 | -3.72203 | 0.000198 |
| **WFDC1** | 2.322193 | 3.719534 | 0.0002 |
| **DEFA4** | 2.451556 | 3.719063 | 0.0002 |
| **GIMAP6** | 0.191871 | -3.71068 | 0.000207 |
| **HMBS** | 3.601786 | 3.705867 | 0.000211 |
| **DAAM2** | 2.456713 | 3.701627 | 0.000214 |
| **EVL** | 0.15436 | -3.66419 | 0.000248 |
| **NAAA** | 0.108785 | -3.61693 | 0.000298 |
| **MPO** | 2.400432 | 3.602679 | 0.000315 |
| **FGL2** | 0.177766 | -3.59522 | 0.000324 |
| **CYTH1** | 0.005236 | -3.59502 | 0.000324 |
| **RNF125** | 0.078538 | -3.57739 | 0.000347 |
| **FAM43A** | 0.110721 | -3.57657 | 0.000348 |
| **IFT20** | 16.25472 | 3.549618 | 0.000386 |
| **LSM1** | 22.13746 | 3.519368 | 0.000433 |
| **SLC36A4** | 11.00369 | 3.514043 | 0.000441 |
| **BTN3A2** | 0.282319 | -3.4906 | 0.000482 |
| **PLEKHA1** | 0.11708 | -3.48765 | 0.000487 |
| **CD74** | 0.164753 | -3.46162 | 0.000537 |
| **POC1B** | 4.955276 | 3.423745 | 0.000618 |
| **C5orf30** | 2.431757 | 3.41786 | 0.000631 |
| **LCK** | 0.27121 | -3.4146 | 0.000639 |
| **TTYH2** | 0.123865 | -3.40561 | 0.00066 |
| **PDZD8** | 5.892166 | 3.401439 | 0.00067 |
| **TGFBI** | 0.362189 | -3.39907 | 0.000676 |
| **FCGR3A** | 0.124607 | -3.36425 | 0.000768 |
| **CDKN2C** | 3.856367 | 3.337268 | 0.000846 |
| **HLA-DRB1** | 0.212131 | -3.31162 | 0.000928 |
| **RAP1GAP** | 1.934668 | 3.295952 | 0.000981 |
| **MYCBP2** | 0.051815 | -3.27466 | 0.001058 |
| **FCER1A** | 0.287949 | -3.25762 | 0.001123 |
| **EMR3** | 0.446816 | -3.2548 | 0.001135 |
| **GYPB** | 2.678252 | 3.25447 | 0.001136 |
| **MAML2** | 0.226604 | -3.2305 | 0.001236 |
| **CA1** | 3.47258 | 3.221374 | 0.001276 |
| **ZBTB38** | 0.101416 | -3.21681 | 0.001296 |
| **ERO1L** | 8.838132 | 3.211961 | 0.001318 |
| **TIAM1** | 0.323735 | -3.19513 | 0.001398 |
| **KLRG1** | 0.255366 | -3.19335 | 0.001406 |
| **SLC7A6** | 0.128542 | -3.16468 | 0.001553 |
| **ANKRD55** | 0.32673 | -3.15501 | 0.001605 |
| **TAP2** | 0.255209 | -3.14733 | 0.001648 |
| **ANKRD9** | 3.255657 | 3.141896 | 0.001679 |
| **CTNNAL1** | 2.29235 | 3.138551 | 0.001698 |
| **GZMH** | 0.50146 | -3.13688 | 0.001708 |
| **HLA-DRA** | 0.146885 | -3.12772 | 0.001762 |
| **CEACAM8** | 2.358029 | 3.123437 | 0.001788 |
| **FKBP1B** | 2.972687 | 3.119518 | 0.001811 |
| **FGFBP2** | 0.32818 | -3.11935 | 0.001813 |
| **ABI3** | 0.218874 | -3.11534 | 0.001837 |
| **CD24** | 2.840549 | 3.089664 | 0.002004 |
| **IRF1** | 0.131445 | -3.08507 | 0.002035 |
| **HES6** | 1.919281 | 3.071029 | 0.002133 |
| **S1PR5** | 0.286346 | -3.06781 | 0.002156 |
| **GPR56** | 0.211156 | -3.06039 | 0.00221 |
| **RNASE3** | 2.739013 | 3.058401 | 0.002225 |
| **EPHX2** | 0.151937 | -3.05386 | 0.002259 |
| **CARD16** | 0.163289 | -3.05143 | 0.002278 |
| **IL27RA** | 0.186673 | -3.04272 | 0.002345 |
| **RPS27L** | 3.658323 | 3.041098 | 0.002357 |
| **CCL5** | 0.247924 | -3.04064 | 0.002361 |
| **CASP5** | 0.430599 | -3.04029 | 0.002363 |
| **NOSIP** | 0.038364 | -3.034 | 0.002413 |
| **TBC1D4** | 0.150481 | -3.02224 | 0.002509 |
| **ARHGEF18** | 0.044032 | -3.00216 | 0.002681 |
| **SPON2** | 0.288298 | -2.99761 | 0.002721 |
| **REPS1** | 0.165264 | -2.99754 | 0.002722 |
| **THBS1** | 2.901131 | 2.991689 | 0.002774 |
| **HLA-DMA** | 0.31956 | -2.98391 | 0.002846 |
| **MAD1L1** | 0.196166 | -2.97777 | 0.002903 |
| **HLA-DPA1** | 0.21062 | -2.97479 | 0.002932 |
| **PPTC7** | 0.212469 | -2.97392 | 0.00294 |
| **LRRC47** | 0.089177 | -2.9736 | 0.002943 |
| **UBAP1** | 15.42947 | 2.973512 | 0.002944 |
| **GGH** | 2.846984 | 2.970221 | 0.002976 |
| **CLIC3** | 0.302378 | -2.96302 | 0.003046 |
| **CCNB2** | 2.532777 | 2.957851 | 0.003098 |
| **ARHGEF3** | 0.120982 | -2.95051 | 0.003173 |
| **DYRK2** | 0.167699 | -2.94539 | 0.003225 |
| **LOC100131541** | 0.391586 | -2.94248 | 0.003256 |
| **CST3** | 0.212845 | -2.9406 | 0.003276 |
| **CHMP7** | 0.055503 | -2.93986 | 0.003284 |
| **CDC42BPA** | 2.905598 | 2.935964 | 0.003325 |
| **CD1D** | 0.254646 | -2.93594 | 0.003325 |
| **LPXN** | 0.161406 | -2.92158 | 0.003483 |
| **NLRP1** | 0.277515 | -2.92049 | 0.003495 |
| **GZMM** | 0.319687 | -2.92025 | 0.003498 |
| **SYNE2** | 0.228122 | -2.91885 | 0.003513 |
| **FBXO21** | 0.170068 | -2.91509 | 0.003556 |
| **TRIT1** | 0.168376 | -2.91168 | 0.003595 |
| **RBX1** | 7.868292 | 2.909614 | 0.003619 |
| **HIST1H1C** | 4.614963 | 2.898509 | 0.003749 |
| **EAF2** | 3.015483 | 2.874867 | 0.004042 |
| **MAN1A1** | 6.016076 | 2.873746 | 0.004056 |
| **C11orf82** | 0.300825 | -2.87305 | 0.004065 |
| **FURIN** | 9.250202 | 2.85188 | 0.004346 |
| **TCTN1** | 0.108088 | -2.83514 | 0.004581 |
| **LDLR** | 3.054616 | 2.833266 | 0.004607 |
| **TTC9** | 0.228477 | -2.83296 | 0.004612 |
| **UBASH3A** | 0.188535 | -2.8291 | 0.004668 |
| **FAM160A2** | 0.125232 | -2.82892 | 0.004671 |
| **ADRB2** | 0.163844 | -2.82727 | 0.004695 |
| **SIAH2** | 4.294978 | 2.826993 | 0.004699 |
| **RARRES3** | 0.399983 | -2.81987 | 0.004804 |
| **ZNF512** | 0.041545 | -2.81236 | 0.004918 |
| **KREMEN1** | 0.313398 | -2.80757 | 0.004992 |
| **PRKCH** | 0.263826 | -2.80009 | 0.005109 |
| **COX7B** | 3.537345 | 2.798996 | 0.005126 |
| **LRRN3** | 0.142344 | -2.79826 | 0.005138 |
| **DPP4** | 0.208986 | -2.79796 | 0.005143 |
| **RNASEN** | 0.121247 | -2.79383 | 0.005209 |
| **GIMAP7** | 0.24483 | -2.78679 | 0.005323 |
| **ZDHHC3** | 12.97848 | 2.785771 | 0.00534 |
| **SEC62** | 5.779478 | 2.776023 | 0.005503 |
| **TPM1** | 3.358704 | 2.774511 | 0.005528 |
| **VSIG4** | 1.823818 | 2.768389 | 0.005633 |
| **HIST2H2BE** | 0.231205 | -2.76632 | 0.005669 |
| **ABCC13** | 1.842424 | 2.765955 | 0.005676 |
| **PCYOX1L** | 0.247522 | -2.76407 | 0.005709 |
| **DPEP2** | 0.404874 | -2.75902 | 0.005797 |
| **F5** | 0.25805 | -2.75218 | 0.00592 |
| **ARG1** | 4.123845 | 2.745792 | 0.006037 |
| **TRBC1** | 0.399141 | -2.74397 | 0.00607 |
| **ENY2** | 6.630758 | 2.740861 | 0.006128 |
| **CD3G** | 0.398784 | -2.72747 | 0.006382 |
| **SPTB** | 2.238553 | 2.726436 | 0.006402 |
| **HBD** | 3.091979 | 2.722948 | 0.00647 |
| **DNAJC9** | 4.304845 | 2.721619 | 0.006496 |
| **RABL2A** | 0.134289 | -2.71503 | 0.006627 |
| **MYCL1** | 0.244466 | -2.71358 | 0.006656 |
| **PROK2** | 0.20618 | -2.70523 | 0.006826 |
| **EOMES** | 0.252456 | -2.70158 | 0.006901 |
| **KLRK1** | 0.397128 | -2.69947 | 0.006945 |
| **CCR3** | 0.398043 | -2.69768 | 0.006983 |
| **NUDT4** | 2.494025 | 2.695412 | 0.00703 |
| **DCUN1D1** | 3.500488 | 2.687238 | 0.007205 |
| **P2RY8** | 0.213906 | -2.68673 | 0.007215 |
| **CD3E** | 0.247055 | -2.68037 | 0.007354 |
| **FCGR1A** | 0.265451 | -2.67421 | 0.007491 |
| **YOD1** | 3.243392 | 2.674026 | 0.007495 |
| **TBX21** | 0.304754 | -2.66902 | 0.007607 |
| **C20orf69** | 0.322019 | -2.66862 | 0.007616 |
| **FLT3LG** | 0.165935 | -2.65985 | 0.007818 |
| **PITPNC1** | 0.175795 | -2.65937 | 0.007829 |
| **GRB10** | 3.552663 | 2.659265 | 0.007831 |
| **SNRNP200** | 0.060233 | -2.65704 | 0.007883 |
| **IL1R2** | 6.784647 | 2.656661 | 0.007892 |
| **CLEC10A** | 0.243604 | -2.65527 | 0.007924 |
| **PQLC3** | 0.314698 | -2.65508 | 0.007929 |
| **ASPH** | 3.161807 | 2.652387 | 0.007992 |
| **TTC39C** | 0.202362 | -2.65187 | 0.008005 |
| **STMN3** | 0.204152 | -2.65154 | 0.008013 |
| **HVCN1** | 0.348835 | -2.64764 | 0.008106 |
| **SKAP1** | 0.248084 | -2.64567 | 0.008153 |
| **SH3YL1** | 0.26448 | -2.64413 | 0.00819 |
| **CCDC23** | 5.382662 | 2.640303 | 0.008283 |
| **PRKCQ** | 0.211022 | -2.63172 | 0.008495 |
| **DHRS3** | 0.305243 | -2.63093 | 0.008515 |
| **MPEG1** | 0.26488 | -2.63072 | 0.00852 |
| **CLSTN1** | 0.132344 | -2.63013 | 0.008535 |
| **DKFZp761E198** | 0.123321 | -2.62296 | 0.008717 |
| **CD2** | 0.443859 | -2.61435 | 0.00894 |
| **TLR5** | 0.28992 | -2.60142 | 0.009284 |
| **FAM113B** | 0.357113 | -2.60082 | 0.0093 |
| **NELL2** | 0.182658 | -2.59809 | 0.009374 |
| **PYHIN1** | 0.15845 | -2.57849 | 0.009923 |
| **BLCAP** | 0.015005 | -2.56928 | 0.010191 |
| **TNFRSF10B** | 0.246581 | -2.56808 | 0.010226 |
| **TTC19** | 0.145245 | -2.56347 | 0.010363 |
| **C2orf89** | 0.282861 | -2.55923 | 0.010491 |
| **NDUFA4** | 7.318054 | 2.554404 | 0.010637 |
| **GPR18** | 0.342468 | -2.55394 | 0.010651 |
| **RFTN1** | 0.170655 | -2.55162 | 0.010722 |
| **RSL24D1** | 2.776132 | 2.551421 | 0.010728 |
| **SH3GLB1** | 0.057038 | -2.55004 | 0.010771 |
| **IFIT1B** | 1.686477 | 2.544985 | 0.010928 |
| **PAFAH2** | 0.109794 | -2.54099 | 0.011054 |
| **CCDC76** | 0.1483 | -2.5362 | 0.011206 |
| **NMT2** | 0.122382 | -2.53065 | 0.011385 |
| **CHPT1** | 8.624673 | 2.528823 | 0.011445 |
| **JAK1** | 0.056662 | -2.52861 | 0.011451 |
| **RNF182** | 1.491798 | 2.520644 | 0.011714 |
| **HLA-DPB1** | 0.293393 | -2.51262 | 0.011984 |
| **PRPF8** | 0.106126 | -2.51205 | 0.012003 |
| **DUSP13** | 2.490176 | 2.505869 | 0.012215 |
| **RNF11** | 2.703075 | 2.501417 | 0.01237 |
| **FOXJ3** | 0.164365 | -2.50124 | 0.012376 |
| **KLRD1** | 0.30634 | -2.49948 | 0.012438 |
| **SLC25A40** | 2.753196 | 2.49839 | 0.012476 |
| **PJA1** | 0.144231 | -2.4966 | 0.012539 |
| **AIM2** | 0.359661 | -2.4965 | 0.012542 |
| **MXI1** | 2.777913 | 2.496493 | 0.012543 |
| **SESN3** | 2.162005 | 2.496421 | 0.012545 |
| **CD96** | 0.171926 | -2.48726 | 0.012873 |
| **SGK1** | 0.418432 | -2.48053 | 0.013119 |
| **FAM117B** | 0.452674 | -2.4805 | 0.01312 |
| **IL32** | 0.396893 | -2.46916 | 0.013543 |
| **TRIB2** | 0.276376 | -2.46478 | 0.01371 |
| **PSMG2** | 0.060948 | -2.46455 | 0.013719 |
| **CD3D** | 0.416735 | -2.4627 | 0.01379 |
| **ARL4C** | 0.363364 | -2.45608 | 0.014046 |
| **PSMA6** | 7.838921 | 2.452865 | 0.014172 |
| **KLF2** | 0.027962 | -2.44896 | 0.014327 |
| **BTN3A1** | 0.25184 | -2.44196 | 0.014608 |
| **ZNF275** | 0.324474 | -2.43826 | 0.014758 |
| **XK** | 2.176532 | 2.437888 | 0.014773 |
| **OXNAD1** | 0.120738 | -2.43474 | 0.014903 |
| **CPEB4** | 7.056265 | 2.430552 | 0.015076 |
| **GIMAP5** | 0.271773 | -2.42467 | 0.015322 |
| **LYSMD2** | 0.393497 | -2.41452 | 0.015756 |
| **UBE2H** | 5.685537 | 2.411049 | 0.015907 |
| **ZNF573** | 0.340828 | -2.40989 | 0.015957 |
| **LDOC1L** | 0.292852 | -2.40794 | 0.016043 |
| **GAL3ST4** | 0.179562 | -2.40629 | 0.016116 |
| **SLC4A7** | 0.215442 | -2.40543 | 0.016154 |
| **XYLT1** | 0.314178 | -2.40424 | 0.016206 |
| **AUTS2** | 0.27021 | -2.40222 | 0.016296 |
| **ISCA1** | 2.265192 | 2.401229 | 0.01634 |
| **OLAH** | 1.656578 | 2.40053 | 0.016371 |
| **TNFSF13B** | 0.179577 | -2.39748 | 0.016508 |
| **LEF1** | 0.284735 | -2.39062 | 0.01682 |
| **PRSS23** | 0.120409 | -2.38484 | 0.017087 |
| **C4orf14** | 0.203658 | -2.38437 | 0.017108 |
| **HLA-DMB** | 0.321577 | -2.38174 | 0.017231 |
| **C14orf43** | 0.28265 | -2.37347 | 0.017622 |
| **SLC14A1** | 1.762955 | 2.366546 | 0.017955 |
| **NIPAL3** | 0.303148 | -2.36636 | 0.017964 |
| **PTCH1** | 0.120016 | -2.36606 | 0.017979 |
| **BPGM** | 1.75421 | 2.356944 | 0.018426 |
| **MATK** | 0.288795 | -2.35369 | 0.018588 |
| **SLC20A1** | 0.114195 | -2.35254 | 0.018646 |
| **SAMD3** | 0.279734 | -2.3518 | 0.018683 |
| **CEACAM21** | 3.823134 | 2.351726 | 0.018687 |
| **NUCKS1** | 8.420825 | 2.351594 | 0.018693 |
| **UTRN** | 0.229185 | -2.35108 | 0.018719 |
| **PSME1** | 0.07178 | -2.35078 | 0.018734 |
| **BCL11B** | 0.482153 | -2.34725 | 0.018912 |
| **TRAF5** | 0.351741 | -2.34661 | 0.018945 |
| **CECR1** | 0.465518 | -2.34492 | 0.019031 |
| **FCGR1B** | 0.447958 | -2.34359 | 0.019099 |
| **DOCK10** | 0.290922 | -2.34158 | 0.019202 |
| **GNLY** | 0.458844 | -2.34129 | 0.019217 |
| **G0S2** | 2.429368 | 2.331473 | 0.019728 |
| **CCR5** | 0.274115 | -2.33079 | 0.019765 |
| **LY86** | 0.468723 | -2.32989 | 0.019812 |
| **NSUN3** | 2.693462 | 2.329209 | 0.019848 |
| **PTGDS** | 0.40736 | -2.32814 | 0.019905 |
| **GBP5** | 0.526808 | -2.32802 | 0.019911 |
| **PPM1K** | 0.317278 | -2.32547 | 0.020047 |
| **GADD45A** | 3.542554 | 2.319105 | 0.020389 |
| **CTSO** | 0.414763 | -2.31829 | 0.020434 |
| **TXK** | 0.301275 | -2.31635 | 0.020539 |
| **KIAA1539** | 7.006559 | 2.313913 | 0.020673 |
| **RUNX3** | 0.280849 | -2.30884 | 0.020952 |
| **M6PR** | 0.104093 | -2.30533 | 0.021148 |
| **CCDC109B** | 0.215673 | -2.30169 | 0.021353 |
| **GATA2** | 0.148729 | -2.30153 | 0.021362 |
| **RASGRP1** | 0.256896 | -2.29701 | 0.021618 |
| **CPVL** | 0.518807 | -2.29571 | 0.021693 |
| **CAMP** | 0.444686 | -2.29107 | 0.021959 |
| **PECAM1** | 0.221808 | -2.2838 | 0.022383 |
| **C11orf46** | 0.144202 | -2.28061 | 0.022572 |
| **CHIT1** | 1.616981 | 2.265064 | 0.023509 |
| **STK39** | 0.205418 | -2.26479 | 0.023526 |
| **PPP4R2** | 4.316647 | 2.263035 | 0.023634 |
| **SMOX** | 2.157896 | 2.26269 | 0.023655 |
| **TNRC6C** | 0.291949 | -2.26249 | 0.023667 |
| **RIOK3** | 3.384871 | 2.261207 | 0.023746 |
| **NFATC3** | 0.260775 | -2.25459 | 0.024159 |
| **SBK1** | 0.235218 | -2.23849 | 0.025189 |
| **TSPAN2** | 3.219654 | 2.234549 | 0.025447 |
| **CISD2** | 2.08763 | 2.23298 | 0.02555 |
| **PTPRCAP** | 0.411198 | -2.23235 | 0.025592 |
| **IER3** | 5.182841 | 2.230365 | 0.025723 |
| **MGA** | 0.21344 | -2.22793 | 0.025885 |
| **AHSP** | 2.291225 | 2.219824 | 0.026431 |
| **CHST7** | 0.433488 | -2.2158 | 0.026705 |
| **RNF44** | 0.193685 | -2.2157 | 0.026712 |
| **IL23A** | 0.352135 | -2.21489 | 0.026768 |
| **BCL2A1** | 3.047735 | 2.210043 | 0.027102 |
| **TRBV9** | 0.376331 | -2.20956 | 0.027136 |
| **COMMD6** | 2.47772 | 2.203331 | 0.027571 |
| **GATA3** | 0.117126 | -2.19943 | 0.027847 |
| **FECH** | 1.799316 | 2.197131 | 0.028011 |
| **TNFRSF25** | 0.198374 | -2.19227 | 0.02836 |
| **HMGB2** | 5.355409 | 2.181493 | 0.029147 |
| **NCRNA00219** | 3.47137 | 2.170973 | 0.029933 |
| **AGTRAP** | 0.10068 | -2.16923 | 0.030065 |
| **BIN1** | 0.242215 | -2.1659 | 0.030319 |
| **FAM78A** | 0.162884 | -2.16069 | 0.030719 |
| **IL4R** | 0.312574 | -2.15767 | 0.030954 |
| **EXOSC10** | 0.301744 | -2.14882 | 0.031649 |
| **IFIT2** | 0.630756 | -2.1424 | 0.032161 |
| **CD81** | 0.355922 | -2.13901 | 0.032435 |
| **SAMSN1** | 3.206046 | 2.126517 | 0.03346 |
| **TGFA** | 0.463543 | -2.1202 | 0.033989 |
| **NSUN5P2** | 0.37042 | -2.11837 | 0.034144 |
| **APOL3** | 0.349048 | -2.10199 | 0.035554 |
| **LYAR** | 0.25272 | -2.09741 | 0.035957 |
| **EIF3D** | 0.047121 | -2.09357 | 0.036298 |
| **CACNA2D3** | 0.335529 | -2.08352 | 0.037203 |
| **UBE2F** | 4.552225 | 2.083482 | 0.037207 |
| **TRAT1** | 0.393366 | -2.07457 | 0.038027 |
| **POM121** | 0.212239 | -2.06588 | 0.03884 |
| **CD8B** | 0.367576 | -2.06559 | 0.038867 |
| **PYGL** | 0.161614 | -2.062 | 0.039208 |
| **ITGAM** | 0.113628 | -2.05662 | 0.039723 |
| **C2orf88** | 2.439574 | 2.05269 | 0.040103 |
| **PAQR8** | 0.177794 | -2.05209 | 0.040161 |
| **NAIP** | 0.377496 | -2.05027 | 0.040338 |
| **PDCD10** | 3.665754 | 2.046482 | 0.040709 |
| **UBE2L6** | 0.32308 | -2.04147 | 0.041204 |
| **ADAM9** | 3.223744 | 2.039412 | 0.041409 |
| **GCLM** | 4.015768 | 2.034204 | 0.041931 |
| **CD6** | 0.336308 | -2.03288 | 0.042065 |
| **MRI1** | 0.229598 | -2.02568 | 0.042798 |
| **CREG1** | 4.124614 | 2.025053 | 0.042862 |
| **LOC100132062** | 0.363785 | -2.02492 | 0.042876 |
| **SIDT1** | 0.317753 | -2.02413 | 0.042956 |
| **AZI2** | 3.303089 | 2.010143 | 0.044416 |
| **LRRC33** | 0.300148 | -2.00199 | 0.045286 |
| **TPST1** | 2.077354 | 1.998461 | 0.045667 |
| **GRAMD1A** | 0.237663 | -1.99209 | 0.046361 |
| **SIRPG** | 0.300269 | -1.99138 | 0.046439 |
| **VNN1** | 2.383725 | 1.982875 | 0.047381 |
| **TFDP1** | 2.059017 | 1.98143 | 0.047543 |
| **IL2RB** | 0.483856 | -1.97947 | 0.047763 |
| **ATP8B4** | 2.02751 | 1.979222 | 0.047791 |
| **CRTC3** | 0.309167 | -1.97597 | 0.048158 |
| **NFATC2** | 0.381874 | -1.9747 | 0.048302 |
| **BTG1** | 0.097921 | -1.97425 | 0.048353 |
| **LGALS2** | 0.609445 | -1.96694 | 0.04919 |
| **GAR1** | 0.304413 | -1.96501 | 0.049413 |

**SM Table 2. Candidate molecules screened by Lasso regression analyses with cross validation.**

| **Genes screen by LASSO**  **EPHX2** |
| --- |
| **LRRN3** |
| **TBC1D4** |
| **CD160** |
| **RABL2B** |
| **DHRS3** |
| **PAFAH2** |
| **SH3GLB1** |
| **PSMG2** |
| **NUCKS1** |
| **TTC9** |
| **PCYOX1L** |
| **CX3CR1** |
| **FAM160A2** |
| **IER3** |
| **PPM1K** |
| **GIMAP4** |
| **ERO1L** |
| **ADRB2** |
| **NCRNA00219** |
| **PDZD8** |
| **HIST1H3D** |
| **RPS27L** |
| **CD1D** |
| **CAMP** |
| **C11orf82** |
| **LDLR** |
| **DAAM2** |
| **ANKRD55** |
| **NME4** |
| **DEFA4** |
| **VSIG4** |
| **RAP1GAP** |
| **KRT23** |

**SM Table 3. Prognostic pathways in sepsis screened by univariate Cox regression**

| **Pathways screened by univariate Cox regression** | **HR** | **z** | **pvalue** |
| --- | --- | --- | --- |
| **REACTOME_IMMUNOREGULATORY_INTERACTIONS_BETWEEN_A_LYMPHOID_AND_A_NON_LYMPHOID_CELL** | 0.396985 | -4.47727 | 7.56E-06 |
| **REACTOME_CD28_DEPENDENT_VAV1_PATHWAY** | 0.514133 | -4.40528 | 1.06E-05 |
| **SA_REG_CASCADE_OF_CYCLIN_EXPR** | 2.572963 | 4.351701 | 1.35E-05 |
| **PID_CD8_TCR_PATHWAY** | 0.395036 | -4.26074 | 2.04E-05 |
| **SA_MMP_CYTOKINE_CONNECTION** | 0.502658 | -4.25791 | 2.06E-05 |
| **PID_HIV_NEF_PATHWAY** | 0.286194 | -4.2265 | 2.37E-05 |
| **BIOCARTA_NKT_PATHWAY** | 0.376618 | -4.22645 | 2.37E-05 |
| **REACTOME_REGULATION_OF_KIT_SIGNALING** | 0.502285 | -4.20712 | 2.59E-05 |
| **KEGG_NITROGEN_METABOLISM** | 3.11882 | 4.194745 | 2.73E-05 |
| **BIOCARTA_DEATH_PATHWAY** | 0.329413 | -4.17571 | 2.97E-05 |
| **PID_TCR_PATHWAY** | 0.372836 | -4.1518 | 3.30E-05 |
| **KEGG_NATURAL_KILLER_CELL_MEDIATED_CYTOTOXICITY** | 0.314292 | -4.14861 | 3.34E-05 |
| **BIOCARTA_P27_PATHWAY** | 2.288355 | 4.094194 | 4.24E-05 |
| **PID_E2F_PATHWAY** | 4.097538 | 4.089218 | 4.33E-05 |
| **BIOCARTA_MCM_PATHWAY** | 2.000188 | 4.080304 | 4.50E-05 |
| **REACTOME_GENERATION_OF_SECOND_MESSENGER_MOLECULES** | 0.581514 | -3.97742 | 6.97E-05 |
| **REACTOME_EXTRINSIC_PATHWAY_FOR_APOPTOSIS** | 0.531904 | -3.90656 | 9.36E-05 |
| **PID_RAC1_REG_PATHWAY** | 0.422565 | -3.8797 | 0.000105 |
| **REACTOME_TCR_SIGNALING** | 0.46931 | -3.84128 | 0.000122 |
| **KEGG_LEISHMANIA_INFECTION** | 0.402977 | -3.80371 | 0.000143 |
| **KEGG_CYTOSOLIC_DNA_SENSING_PATHWAY** | 0.380758 | -3.76879 | 0.000164 |
| **REACTOME_PHOSPHORYLATION_OF_CD3_AND_TCR_ZETA_CHAINS** | 0.604615 | -3.73767 | 0.000186 |
| **BIOCARTA_IL7_PATHWAY** | 0.529721 | -3.71759 | 0.000201 |
| **ST_T_CELL_SIGNAL_TRANSDUCTION** | 0.450595 | -3.67946 | 0.000234 |
| **REACTOME_PD1_SIGNALING** | 0.609183 | -3.65927 | 0.000253 |
| **BIOCARTA_DC_PATHWAY** | 0.489876 | -3.64091 | 0.000272 |
| **REACTOME_SIGNAL_REGULATORY_PROTEIN_SIRP_FAMILY_INTERACTIONS** | 0.559736 | -3.61199 | 0.000304 |
| **REACTOME_IMMUNE_SYSTEM** | 0.116217 | -3.60721 | 0.00031 |
| **REACTOME_G0_AND_EARLY_G1** | 2.432835 | 3.599375 | 0.000319 |
| **REACTOME_COSTIMULATION_BY_THE_CD28_FAMILY** | 0.351323 | -3.5649 | 0.000364 |
| **BIOCARTA_THELPER_PATHWAY** | 0.610964 | -3.55429 | 0.000379 |
| **REACTOME_SYNTHESIS_AND_INTERCONVERSION_OF_NUCLEOTIDE_DI_AND_TRIPHOSPHATES** | 2.105201 | 3.494613 | 0.000475 |
| **PID_FOXM1_PATHWAY** | 2.364688 | 3.446831 | 0.000567 |
| **REACTOME_NFKB_ACTIVATION_THROUGH_FADD_RIP1_PATHWAY_MEDIATED_BY_CASPASE_8_AND10** | 0.598141 | -3.43868 | 0.000585 |
| **KEGG_HEMATOPOIETIC_CELL_LINEAGE** | 0.344092 | -3.42864 | 0.000607 |
| **PID_IL27_PATHWAY** | 0.497485 | -3.40421 | 0.000664 |
| **BIOCARTA_RELA_PATHWAY** | 0.58339 | -3.40032 | 0.000673 |
| **PID_PLK1_PATHWAY** | 2.289242 | 3.365557 | 0.000764 |
| **REACTOME_TRANSPORT_OF_GLUCOSE_AND_OTHER_SUGARS_BILE_SALTS_AND_ORGANIC_ACIDS_METAL_IONS_AND_AMINE_COMPOUNDS** | 2.910501 | 3.361566 | 0.000775 |
| **REACTOME_DOWNSTREAM_TCR_SIGNALING** | 0.528204 | -3.35755 | 0.000786 |
| **PID_P38_ALPHA_BETA_PATHWAY** | 0.455696 | -3.3564 | 0.00079 |
| **BIOCARTA_MONOCYTE_PATHWAY** | 0.581487 | -3.3073 | 0.000942 |
| **BIOCARTA_CASPASE_PATHWAY** | 0.422134 | -3.30325 | 0.000956 |
| **PID_PI3KCI_PATHWAY** | 0.434429 | -3.29776 | 0.000975 |
| **REACTOME_AMINE_COMPOUND_SLC_TRANSPORTERS** | 1.657032 | 3.295681 | 0.000982 |
| **REACTOME_G1_S_SPECIFIC_TRANSCRIPTION** | 1.649635 | 3.277401 | 0.001048 |
| **BIOCARTA_CSK_PATHWAY** | 0.56545 | -3.27131 | 0.00107 |
| **REACTOME_ACTIVATION_OF_THE_PRE_REPLICATIVE_COMPLEX** | 1.849677 | 3.26884 | 0.00108 |
| **BIOCARTA_TCYTOTOXIC_PATHWAY** | 0.644929 | -3.24174 | 0.001188 |
| **PID_ATF2_PATHWAY** | 3.013639 | 3.229697 | 0.001239 |
| **REACTOME_PKB_MEDIATED_EVENTS** | 3.08723 | 3.204095 | 0.001355 |
| **BIOCARTA_GRANULOCYTES_PATHWAY** | 0.563376 | -3.20401 | 0.001355 |
| **KEGG_PRIMARY_IMMUNODEFICIENCY** | 0.491985 | -3.18853 | 0.00143 |
| **BIOCARTA_TRKA_PATHWAY** | 0.572913 | -3.18752 | 0.001435 |
| **KEGG_ASTHMA** | 0.596336 | -3.17124 | 0.001518 |
| **BIOCARTA_IL10_PATHWAY** | 0.522772 | -3.1702 | 0.001523 |
| **PID_PRL_SIGNALING_EVENTS_PATHWAY** | 2.418217 | 3.14653 | 0.001652 |
| **KEGG_VIRAL_MYOCARDITIS** | 0.495325 | -3.13996 | 0.00169 |
| **BIOCARTA_AHSP_PATHWAY** | 1.508939 | 3.119163 | 0.001814 |
| **BIOCARTA_NFKB_PATHWAY** | 0.552775 | -3.11766 | 0.001823 |
| **REACTOME_DAG_AND_IP3_SIGNALING** | 0.398246 | -3.11673 | 0.001829 |
| **KEGG_GRAFT_VERSUS_HOST_DISEASE** | 0.64681 | -3.11267 | 0.001854 |
| **REACTOME_ADAPTIVE_IMMUNE_SYSTEM** | 0.121023 | -3.11184 | 0.001859 |
| **KEGG_PROXIMAL_TUBULE_BICARBONATE_RECLAMATION** | 2.206918 | 3.10963 | 0.001873 |
| **REACTOME_TRANSLOCATION_OF_ZAP_70_TO_IMMUNOLOGICAL_SYNAPSE** | 0.730212 | -3.08494 | 0.002036 |
| **BIOCARTA_HIVNEF_PATHWAY** | 0.39927 | -3.07252 | 0.002123 |
| **REACTOME_P75_NTR_RECEPTOR_MEDIATED_SIGNALLING** | 0.359434 | -3.07067 | 0.002136 |
| **REACTOME_BIOLOGICAL_OXIDATIONS** | 3.335608 | 3.069332 | 0.002145 |
| **SA_G1_AND_S_PHASES** | 2.196944 | 3.069052 | 0.002147 |
| **REACTOME_PI3K_CASCADE** | 3.573269 | 3.027833 | 0.002463 |
| **PID_TRAIL_PATHWAY** | 0.503577 | -3.02188 | 0.002512 |
| **BIOCARTA_NO2IL12_PATHWAY** | 0.662565 | -3.02087 | 0.002521 |
| **PID_FCER1_PATHWAY** | 0.481063 | -3.01329 | 0.002584 |
| **KEGG_ALLOGRAFT_REJECTION** | 0.626933 | -3.01049 | 0.002608 |
| **BIOCARTA_TCRA_PATHWAY** | 0.712067 | -3.01008 | 0.002612 |
| **BIOCARTA_CBL_PATHWAY** | 0.613181 | -3.01003 | 0.002612 |
| **REACTOME_TRAF6_MEDIATED_IRF7_ACTIVATION** | 0.610988 | -3.00928 | 0.002619 |
| **REACTOME_DIABETES_PATHWAYS** | 3.75728 | 3.004315 | 0.002662 |
| **BIOCARTA_HIF_PATHWAY** | 1.882547 | 2.980539 | 0.002877 |
| **REACTOME_IL_3_5_AND_GM_CSF_SIGNALING** | 0.513622 | -2.97995 | 0.002883 |
| **KEGG_GLYCOSPHINGOLIPID_BIOSYNTHESIS_LACTO_AND_NEOLACTO_SERIES** | 1.941412 | 2.972451 | 0.002954 |
| **PID_HIF2PATHWAY** | 3.06663 | 2.969635 | 0.002982 |
| **KEGG_BLADDER_CANCER** | 2.966298 | 2.947861 | 0.0032 |
| **KEGG_T_CELL_RECEPTOR_SIGNALING_PATHWAY** | 0.417068 | -2.94189 | 0.003262 |
| **REACTOME_CA_DEPENDENT_EVENTS** | 0.468461 | -2.90204 | 0.003707 |
| **REACTOME_INTERFERON_GAMMA_SIGNALING** | 0.578568 | -2.89247 | 0.003822 |
| **REACTOME_ACTIVATION_OF_CHAPERONES_BY_ATF6_ALPHA** | 1.747089 | 2.878921 | 0.00399 |
| **REACTOME_SLC_MEDIATED_TRANSMEMBRANE_TRANSPORT** | 4.591594 | 2.878158 | 0.004 |
| **BIOCARTA_TNFR1_PATHWAY** | 0.472292 | -2.86497 | 0.004171 |
| **REACTOME_CELL_DEATH_SIGNALLING_VIA_NRAGE_NRIF_AND_NADE** | 0.39417 | -2.86361 | 0.004188 |
| **BIOCARTA_CREB_PATHWAY** | 0.513187 | -2.86276 | 0.0042 |
| **REACTOME_E2F_MEDIATED_REGULATION_OF_DNA_REPLICATION** | 1.677705 | 2.849313 | 0.004381 |
| **REACTOME_INTERFERON_SIGNALING** | 0.504253 | -2.8484 | 0.004394 |
| **KEGG_APOPTOSIS** | 0.364494 | -2.83052 | 0.004647 |
| **REACTOME_TRAF3_DEPENDENT_IRF_ACTIVATION_PATHWAY** | 0.604446 | -2.82247 | 0.004766 |
| **PID_HEDGEHOG_GLI_PATHWAY** | 0.42844 | -2.82052 | 0.004795 |
| **REACTOME_CIRCADIAN_REPRESSION_OF_EXPRESSION_BY_REV_ERBA** | 0.597194 | -2.79908 | 0.005125 |
| **KEGG_ARGININE_AND_PROLINE_METABOLISM** | 2.377679 | 2.79111 | 0.005253 |
| **BIOCARTA_TID_PATHWAY** | 0.593389 | -2.78692 | 0.005321 |
| **KEGG_ANTIGEN_PROCESSING_AND_PRESENTATION** | 0.591531 | -2.7842 | 0.005366 |
| **REACTOME_SYNTHESIS_OF_PC** | 2.251843 | 2.771097 | 0.005587 |
| **BIOCARTA_CTL_PATHWAY** | 0.669162 | -2.77015 | 0.005603 |
| **REACTOME_CYTOKINE_SIGNALING_IN_IMMUNE_SYSTEM** | 0.442815 | -2.75786 | 0.005818 |
| **BIOCARTA_TH1TH2_PATHWAY** | 0.543514 | -2.7561 | 0.00585 |
| **REACTOME_KINESINS** | 1.839835 | 2.749414 | 0.00597 |
| **ST_INTERLEUKIN_4_PATHWAY** | 0.513224 | -2.73433 | 0.006251 |
| **BIOCARTA_CELLCYCLE_PATHWAY** | 1.871586 | 2.727432 | 0.006383 |
| **REACTOME_ACTIVATED_NOTCH1_TRANSMITS_SIGNAL_TO_THE_NUCLEUS** | 0.537733 | -2.71485 | 0.006631 |
| **BIOCARTA_FAS_PATHWAY** | 0.522127 | -2.71484 | 0.006631 |
| **BIOCARTA_BIOPEPTIDES_PATHWAY** | 0.464179 | -2.71408 | 0.006646 |
| **REACTOME_AMINO_ACID_TRANSPORT_ACROSS_THE_PLASMA_MEMBRANE** | 1.852858 | 2.692572 | 0.00709 |
| **REACTOME_PI3K_AKT_ACTIVATION** | 2.318322 | 2.691117 | 0.007121 |
| **BIOCARTA_GSK3_PATHWAY** | 0.545003 | -2.6908 | 0.007128 |
| **BIOCARTA_IL2RB_PATHWAY** | 0.48432 | -2.6777 | 0.007413 |
| **REACTOME_RORA_ACTIVATES_CIRCADIAN_EXPRESSION** | 0.589991 | -2.67577 | 0.007456 |
| **KEGG_P53_SIGNALING_PATHWAY** | 2.686473 | 2.669098 | 0.007606 |
| **REACTOME_ACTIVATION_OF_ATR_IN_RESPONSE_TO_REPLICATION_STRESS** | 1.689836 | 2.665173 | 0.007695 |
| **REACTOME_G2_M_CHECKPOINTS** | 1.654663 | 2.653634 | 0.007963 |
| **KEGG_AUTOIMMUNE_THYROID_DISEASE** | 0.671397 | -2.65025 | 0.008043 |
| **BIOCARTA_TEL_PATHWAY** | 0.534986 | -2.6495 | 0.008061 |
| **REACTOME_THE_ROLE_OF_NEF_IN_HIV1_REPLICATION_AND_DISEASE_PATHOGENESIS** | 0.558029 | -2.64824 | 0.008091 |
| **BIOCARTA_G2_PATHWAY** | 2.120388 | 2.640769 | 0.008272 |
| **REACTOME_PKA_MEDIATED_PHOSPHORYLATION_OF_CREB** | 0.557661 | -2.63343 | 0.008453 |
| **REACTOME_PI_3K_CASCADE** | 2.363808 | 2.62629 | 0.008632 |
| **REACTOME_PI3K_EVENTS_IN_ERBB4_SIGNALING** | 2.390844 | 2.622998 | 0.008716 |
| **SIG_BCR_SIGNALING_PATHWAY** | 0.529203 | -2.61754 | 0.008857 |
| **REACTOME_REGULATORY_RNA_PATHWAYS** | 0.523526 | -2.61463 | 0.008932 |
| **PID_P73PATHWAY** | 3.761883 | 2.611353 | 0.009018 |
| **REACTOME_AMINO_ACID_AND_OLIGOPEPTIDE_SLC_TRANSPORTERS** | 2.027856 | 2.610696 | 0.009036 |
| **BIOCARTA_TCAPOPTOSIS_PATHWAY** | 0.713046 | -2.60542 | 0.009176 |
| **BIOCARTA_TCR_PATHWAY** | 0.504309 | -2.57937 | 0.009898 |
| **KEGG_CARDIAC_MUSCLE_CONTRACTION** | 1.831405 | 2.569736 | 0.010178 |
| **REACTOME_DNA_STRAND_ELONGATION** | 1.587844 | 2.567171 | 0.010253 |
| **KEGG_CELL_CYCLE** | 2.222696 | 2.553327 | 0.01067 |
| **PID_HIF1_TFPATHWAY** | 2.358033 | 2.531907 | 0.011344 |
| **REACTOME_IL_RECEPTOR_SHC_SIGNALING** | 0.575841 | -2.52017 | 0.01173 |
| **REACTOME_INFLAMMASOMES** | 0.56349 | -2.51701 | 0.011835 |
| **KEGG_MTOR_SIGNALING_PATHWAY** | 2.512141 | 2.508262 | 0.012133 |
| **REACTOME_EFFECTS_OF_PIP2_HYDROLYSIS** | 0.643034 | -2.50459 | 0.012259 |
| **REACTOME_G1_PHASE** | 2.219033 | 2.494336 | 0.012619 |
| **SA_PTEN_PATHWAY** | 0.623123 | -2.49157 | 0.012718 |
| **BIOCARTA_IL3_PATHWAY** | 0.644794 | -2.48982 | 0.012781 |
| **REACTOME_INHIBITION_OF_REPLICATION_INITIATION_OF_DAMAGED_DNA_BY_RB1_E2F1** | 1.737138 | 2.48033 | 0.013126 |
| **REACTOME_ANTIGEN_ACTIVATES_B_CELL_RECEPTOR_LEADING_TO_GENERATION_OF_SECOND_MESSENGERS** | 0.589254 | -2.46782 | 0.013594 |
| **PID_IL12_2PATHWAY** | 0.52596 | -2.46263 | 0.013792 |
| **KEGG_TYPE_I_DIABETES_MELLITUS** | 0.675877 | -2.44009 | 0.014684 |
| **REACTOME_ENERGY_DEPENDENT_REGULATION_OF_MTOR_BY_LKB1_AMPK** | 1.734305 | 2.438837 | 0.014735 |
| **BIOCARTA_TOB1_PATHWAY** | 0.636264 | -2.43373 | 0.014944 |
| **REACTOME_CYCLIN_A_B1_ASSOCIATED_EVENTS_DURING_G2_M_TRANSITION** | 1.470404 | 2.42437 | 0.015335 |
| **BIOCARTA_TOLL_PATHWAY** | 0.543984 | -2.42086 | 0.015484 |
| **REACTOME_TRANSMEMBRANE_TRANSPORT_OF_SMALL_MOLECULES** | 4.260295 | 2.415898 | 0.015696 |
| **KEGG_SYSTEMIC_LUPUS_ERYTHEMATOSUS** | 0.483251 | -2.41568 | 0.015706 |
| **KEGG_BUTANOATE_METABOLISM** | 1.828615 | 2.41122 | 0.015899 |
| **PID_CASPASE_PATHWAY** | 0.406636 | -2.40919 | 0.015988 |
| **PID_EPHRINB_REV_PATHWAY** | 0.556447 | -2.40521 | 0.016163 |
| **PID_EPO_PATHWAY** | 0.578329 | -2.40424 | 0.016206 |
| **REACTOME_INSULIN_RECEPTOR_SIGNALLING_CASCADE** | 2.729266 | 2.400326 | 0.01638 |
| **REACTOME_THE_NLRP3_INFLAMMASOME** | 0.631066 | -2.39982 | 0.016403 |
| **KEGG_CYTOKINE_CYTOKINE_RECEPTOR_INTERACTION** | 0.441091 | -2.39965 | 0.016411 |
| **REACTOME_DEGRADATION_OF_THE_EXTRACELLULAR_MATRIX** | 1.59871 | 2.396899 | 0.016534 |
| **REACTOME_REGULATION_OF_AMPK_ACTIVITY_VIA_LKB1** | 1.599128 | 2.393766 | 0.016676 |
| **KEGG_NOD_LIKE_RECEPTOR_SIGNALING_PATHWAY** | 0.529835 | -2.39371 | 0.016679 |
| **KEGG_STEROID_BIOSYNTHESIS** | 1.656283 | 2.390403 | 0.01683 |
| **REACTOME_PROCESSING_OF_INTRONLESS_PRE_MRNAS** | 0.697129 | -2.38415 | 0.017119 |
| **REACTOME_METABOLISM_OF_NUCLEOTIDES** | 2.259304 | 2.381167 | 0.017258 |
| **SIG_PIP3_SIGNALING_IN_B_LYMPHOCYTES** | 0.591624 | -2.38055 | 0.017287 |
| **PID_ANGIOPOIETIN_RECEPTOR_PATHWAY** | 0.53977 | -2.37743 | 0.017434 |
| **BIOCARTA_AGPCR_PATHWAY** | 0.672006 | -2.37419 | 0.017587 |
| **REACTOME_NEUROTRANSMITTER_RELEASE_CYCLE** | 1.771865 | 2.371518 | 0.017715 |
| **REACTOME_PHASE_II_CONJUGATION** | 2.002001 | 2.369073 | 0.017833 |
| **REACTOME_BASIGIN_INTERACTIONS** | 1.73454 | 2.347052 | 0.018923 |
| **REACTOME_CD28_CO_STIMULATION** | 0.515381 | -2.34512 | 0.019021 |
| **KEGG_MELANOMA** | 2.173707 | 2.341742 | 0.019194 |
| **PID_AP1_PATHWAY** | 2.170875 | 2.337616 | 0.019407 |
| **PID_IL2_STAT5_PATHWAY** | 0.56552 | -2.33656 | 0.019462 |
| **BIOCARTA_RANKL_PATHWAY** | 0.73189 | -2.32791 | 0.019917 |
| **PID_BCR_5PATHWAY** | 0.544707 | -2.32621 | 0.020007 |
| **BIOCARTA_CHEMICAL_PATHWAY** | 0.507396 | -2.32187 | 0.02024 |
| **REACTOME_CTLA4_INHIBITORY_SIGNALING** | 0.548834 | -2.32038 | 0.02032 |
| **REACTOME_GENERIC_TRANSCRIPTION_PATHWAY** | 0.425444 | -2.31457 | 0.020636 |
| **KEGG_RIG_I_LIKE_RECEPTOR_SIGNALING_PATHWAY** | 0.539722 | -2.30962 | 0.020909 |
| **BIOCARTA_KERATINOCYTE_PATHWAY** | 0.532789 | -2.30412 | 0.021216 |
| **REACTOME_PI3K_EVENTS_IN_ERBB2_SIGNALING** | 2.139666 | 2.299227 | 0.021492 |
| **REACTOME_IL_2_SIGNALING** | 0.562214 | -2.29911 | 0.021499 |
| **KEGG_INTESTINAL_IMMUNE_NETWORK_FOR_IGA_PRODUCTION** | 0.652634 | -2.29908 | 0.021501 |
| **REACTOME_MYOGENESIS** | 1.799461 | 2.29903 | 0.021503 |
| **BIOCARTA_MITOCHONDRIA_PATHWAY** | 0.51743 | -2.29512 | 0.021726 |
| **PID_NFKAPPAB_CANONICAL_PATHWAY** | 0.635264 | -2.2912 | 0.021952 |
| **KEGG_NOTCH_SIGNALING_PATHWAY** | 0.570354 | -2.28964 | 0.022042 |
| **REACTOME_TRAFFICKING_AND_PROCESSING_OF_ENDOSOMAL_TLR** | 0.63288 | -2.28882 | 0.02209 |
| **KEGG_CHEMOKINE_SIGNALING_PATHWAY** | 0.529705 | -2.28624 | 0.02224 |
| **PID_EPHA2_FWD_PATHWAY** | 0.605897 | -2.26474 | 0.023529 |
| **REACTOME_ABORTIVE_ELONGATION_OF_HIV1_TRANSCRIPT_IN_THE_ABSENCE_OF_TAT** | 0.576757 | -2.24875 | 0.024528 |
| **REACTOME_RIG_I_MDA5_MEDIATED_INDUCTION_OF_IFN_ALPHA_BETA_PATHWAYS** | 0.545701 | -2.24397 | 0.024835 |
| **REACTOME_TRANSCRIPTIONAL_REGULATION_OF_WHITE_ADIPOCYTE_DIFFERENTIATION** | 0.482169 | -2.24074 | 0.025043 |
| **KEGG_GLYCINE_SERINE_AND_THREONINE_METABOLISM** | 1.801702 | 2.239748 | 0.025107 |
| **PID_LYMPH_ANGIOGENESIS_PATHWAY** | 0.63536 | -2.23946 | 0.025126 |
| **REACTOME_MRNA_DECAY_BY_5_TO_3_EXORIBONUCLEASE** | 1.725236 | 2.238872 | 0.025164 |
| **BIOCARTA_TALL1_PATHWAY** | 0.609778 | -2.23069 | 0.025702 |
| **PID_ARF6_PATHWAY** | 0.576088 | -2.2298 | 0.025761 |
| **REACTOME_INNATE_IMMUNE_SYSTEM** | 0.44863 | -2.22829 | 0.025861 |
| **BIOCARTA_RB_PATHWAY** | 1.634391 | 2.21435 | 0.026805 |
| **SA_CASPASE_CASCADE** | 0.583489 | -2.21379 | 0.026843 |
| **BIOCARTA_CTLA4_PATHWAY** | 0.698263 | -2.20849 | 0.02721 |
| **REACTOME_PHASE1_FUNCTIONALIZATION_OF_COMPOUNDS** | 1.846925 | 2.201751 | 0.027683 |
| **REACTOME_MICRORNA_MIRNA_BIOGENESIS** | 0.568199 | -2.19988 | 0.027815 |
| **KEGG_B_CELL_RECEPTOR_SIGNALING_PATHWAY** | 0.542057 | -2.19521 | 0.028148 |
| **REACTOME_OXYGEN_DEPENDENT_PROLINE_HYDROXYLATION_OF_HYPOXIA_INDUCIBLE_FACTOR_ALPHA** | 1.748694 | 2.190748 | 0.02847 |
| **PID_AURORA_B_PATHWAY** | 1.516098 | 2.189645 | 0.02855 |
| **REACTOME_TGF_BETA_RECEPTOR_SIGNALING_ACTIVATES_SMADS** | 1.917731 | 2.187215 | 0.028727 |
| **REACTOME_UNFOLDED_PROTEIN_RESPONSE** | 2.068594 | 2.183546 | 0.028996 |
| **BIOCARTA_NKCELLS_PATHWAY** | 0.617497 | -2.1827 | 0.029058 |
| **PID_HNF3B_PATHWAY** | 2.184184 | 2.182277 | 0.029089 |
| **REACTOME_INTERFERON_ALPHA_BETA_SIGNALING** | 0.720276 | -2.18181 | 0.029123 |
| **REACTOME_CHOLESTEROL_BIOSYNTHESIS** | 1.648116 | 2.171466 | 0.029896 |
| **BIOCARTA_CERAMIDE_PATHWAY** | 0.593729 | -2.16776 | 0.030177 |
| **REACTOME_NEGATIVE_REGULATORS_OF_RIG_I_MDA5_SIGNALING** | 0.557622 | -2.16637 | 0.030283 |
| **REACTOME_BMAL1_CLOCK_NPAS2_ACTIVATES_CIRCADIAN_EXPRESSION** | 0.61652 | -2.16465 | 0.030415 |
| **BIOCARTA_ERK5_PATHWAY** | 0.669612 | -2.16414 | 0.030454 |
| **PID_RAS_PATHWAY** | 0.569246 | -2.15836 | 0.0309 |
| **REACTOME_LIPID_DIGESTION_MOBILIZATION_AND_TRANSPORT** | 1.899683 | 2.15683 | 0.031019 |
| **REACTOME_REGULATED_PROTEOLYSIS_OF_P75NTR** | 0.713638 | -2.15146 | 0.03144 |
| **BIOCARTA_CACAM_PATHWAY** | 0.646142 | -2.14906 | 0.031629 |
| **REACTOME_ASSOCIATION_OF_TRIC_CCT_WITH_TARGET_PROTEINS_DURING_BIOSYNTHESIS** | 0.561236 | -2.14301 | 0.032113 |
| **REACTOME_NRAGE_SIGNALS_DEATH_THROUGH_JNK** | 0.562112 | -2.132 | 0.033006 |
| **REACTOME_ION_CHANNEL_TRANSPORT** | 1.812576 | 2.130753 | 0.033109 |
| **PID_TCR_RAS_PATHWAY** | 1.741903 | 2.126527 | 0.033459 |
| **KEGG_CELL_ADHESION_MOLECULES_CAMS** | 0.579367 | -2.10381 | 0.035395 |
| **ST_INTEGRIN_SIGNALING_PATHWAY** | 0.519114 | -2.09562 | 0.036116 |
| **BIOCARTA_FCER1_PATHWAY** | 0.579875 | -2.09493 | 0.036177 |
| **REACTOME_ION_TRANSPORT_BY_P_TYPE_ATPASES** | 1.669092 | 2.092698 | 0.036376 |
| **REACTOME_VIRAL_MESSENGER_RNA_SYNTHESIS** | 0.641851 | -2.0864 | 0.036943 |
| **BIOCARTA_IL22BP_PATHWAY** | 0.697386 | -2.08369 | 0.037188 |
| **REACTOME_AKT_PHOSPHORYLATES_TARGETS_IN_THE_CYTOSOL** | 1.633195 | 2.075205 | 0.037968 |
| **BIOCARTA_IL12_PATHWAY** | 0.612189 | -2.06579 | 0.038848 |
| **PID_INTEGRIN2_PATHWAY** | 0.696126 | -2.06525 | 0.038899 |
| **REACTOME_FORMATION_OF_THE_HIV1_EARLY_ELONGATION_COMPLEX** | 0.57585 | -2.06383 | 0.039033 |
| **BIOCARTA_RARRXR_PATHWAY** | 0.643998 | -2.06343 | 0.039071 |
| **BIOCARTA_PITX2_PATHWAY** | 0.697761 | -2.06128 | 0.039276 |
| **BIOCARTA_BCR_PATHWAY** | 0.584285 | -2.05814 | 0.039577 |
| **REACTOME_MRNA_3_END_PROCESSING** | 0.659381 | -2.05729 | 0.039658 |
| **REACTOME_METABOLISM_OF_PORPHYRINS** | 1.381142 | 2.056559 | 0.039729 |
| **BIOCARTA_P53_PATHWAY** | 1.869192 | 2.05514 | 0.039865 |
| **REACTOME_MRNA_CAPPING** | 0.612467 | -2.0454 | 0.040815 |
| **REACTOME_INTEGRIN_ALPHAIIB_BETA3_SIGNALING** | 0.667459 | -2.04296 | 0.041056 |
| **BIOCARTA_BARRESTIN_SRC_PATHWAY** | 0.664262 | -2.0387 | 0.04148 |
| **KEGG_JAK_STAT_SIGNALING_PATHWAY** | 0.525336 | -2.03285 | 0.042068 |
| **PID_FRA_PATHWAY** | 1.767131 | 2.030103 | 0.042346 |
| **KEGG_BIOSYNTHESIS_OF_UNSATURATED_FATTY_ACIDS** | 1.676696 | 2.020452 | 0.043337 |
| **PID_P53_DOWNSTREAM_PATHWAY** | 2.606031 | 2.017166 | 0.043678 |
| **REACTOME_PYRUVATE_METABOLISM** | 1.563683 | 2.016347 | 0.043764 |
| **PID_CXCR4_PATHWAY** | 0.538314 | -2.01251 | 0.044166 |
| **REACTOME_PIP3_ACTIVATES_AKT_SIGNALING** | 1.769772 | 2.008293 | 0.044612 |
| **PID_RXR_VDR_PATHWAY** | 0.664783 | -2.00276 | 0.045203 |
| **REACTOME_GLYCEROPHOSPHOLIPID_BIOSYNTHESIS** | 2.289139 | 1.997237 | 0.045799 |
| **REACTOME_GAB1_SIGNALOSOME** | 1.90364 | 1.99579 | 0.045957 |
| **BIOCARTA_LAIR_PATHWAY** | 0.690507 | -1.99386 | 0.046167 |
| **REACTOME_MITOTIC_G1_G1_S_PHASES** | 1.654902 | 1.991731 | 0.046401 |
| **REACTOME_N_GLYCAN_ANTENNAE_ELONGATION_IN_THE_MEDIAL_TRANS_GOLGI** | 1.619386 | 1.983099 | 0.047356 |
| **BIOCARTA_IL2_PATHWAY** | 0.634296 | -1.981 | 0.047592 |
| **BIOCARTA_PYK2_PATHWAY** | 0.610663 | -1.97746 | 0.04799 |
| **REACTOME_INTERACTION_BETWEEN_L1_AND_ANKYRINS** | 1.361749 | 1.967311 | 0.049147 |
| **PID_MYC_ACTIV_PATHWAY** | 1.71195 | 1.966821 | 0.049204 |
| **KEGG_GLYCOLYSIS_GLUCONEOGENESIS** | 1.629073 | 1.964618 | 0.049458 |
| **BIOCARTA_ACTINY_PATHWAY** | 0.682081 | -1.96339 | 0.049601 |
| **REACTOME_SIGNALING_BY_FGFR1_FUSION_MUTANTS** | 0.672576 | -1.96328 | 0.049614 |
| **BIOCARTA_ARENRF2_PATHWAY** | 1.648545 | 1.962092 | 0.049752 |

**SM Table 4. Pathways in sepsis screened by LASSO regression**

| **Pathway screened by LASSO**  **KEGG_STEROID_BIOSYNTHESIS** |
| --- |
| **KEGG_NITROGEN_METABOLISM** |
| **KEGG_CARDIAC_MUSCLE_CONTRACTION** |
| **KEGG_CYTOSOLIC_DNA_SENSING_PATHWAY** |
| **KEGG_SYSTEMIC_LUPUS_ERYTHEMATOSUS** |
| **BIOCARTA_HIF_PATHWAY** |
| **BIOCARTA_IL22BP_PATHWAY** |
| **BIOCARTA_BARRESTIN_SRC_PATHWAY** |
| **BIOCARTA_NKT_PATHWAY** |
| **BIOCARTA_TEL_PATHWAY** |
| **ST_INTERLEUKIN_4_PATHWAY** |
| **SA_MMP_CYTOKINE_CONNECTION** |
| **SA_REG_CASCADE_OF_CYCLIN_EXPR** |
| **PID_PRL_SIGNALING_EVENTS_PATHWAY** |
| **PID_NFKAPPAB_CANONICAL_PATHWAY** |
| **PID_FRA_PATHWAY** |
| **PID_ARF6_PATHWAY** |
| **PID_ATF2_PATHWAY** |
| **PID_HIV_NEF_PATHWAY** |
| **PID_RAC1_REG_PATHWAY** |
| **REACTOME_PROCESSING_OF_INTRONLESS_PRE_MRNAS** |
| **REACTOME_TGF_BETA_RECEPTOR_SIGNALING_ACTIVATES_SMADS** |
| **REACTOME_MICRORNA_MIRNA_BIOGENESIS** |
| **REACTOME_DOWNSTREAM_TCR_SIGNALING** |
| **REACTOME_GAB1_SIGNALOSOME** |
| **REACTOME_BIOLOGICAL_OXIDATIONS** |
| **REACTOME_CA_DEPENDENT_EVENTS** |
| **REACTOME_PKA_MEDIATED_PHOSPHORYLATION_OF_CREB** |
| **REACTOME_ASSOCIATION_OF_TRIC_CCT_WITH_TARGET_PROTEINS_DURING_BIOSYNTHESIS** |
| **REACTOME_ACTIVATION_OF_CHAPERONES_BY_ATF6_ALPHA** |
| **REACTOME_TRANSPORT_OF_GLUCOSE_AND_OTHER_SUGARS_BILE_SALTS_AND_ORGANIC_ACIDS_METAL_IONS_AND_AMINE_COMPOUNDS** |
| **REACTOME_MRNA_DECAY_BY_5_TO_3_EXORIBONUCLEASE** |
| **REACTOME_MYOGENESIS** |
| **REACTOME_ENERGY_DEPENDENT_REGULATION_OF_MTOR_BY_LKB1_AMPK** |
| **REACTOME_NFKB_ACTIVATION_THROUGH_FADD_RIP1_PATHWAY_MEDIATED_BY_CASPASE_8_AND10** |
| **REACTOME_NEGATIVE_REGULATORS_OF_RIG_I_MDA5_SIGNALING** |
| **REACTOME_ABORTIVE_ELONGATION_OF_HIV1_TRANSCRIPT_IN_THE_ABSENCE_OF_TAT** |

##The code of differential analysis

logFoldChange=1

adjustP=0.05

library(limma)

setwd("D:\\immunity\\COX3\\limma")

rt=read.table("data.txt",sep="\t",header=T,check.names=F)

rt=as.matrix(rt)

rownames(rt)=rt[,1]

exp=rt[,2:ncol(rt)]

dimnames=list(rownames(exp),colnames(exp))

rt=matrix(as.numeric(as.matrix(exp)),nrow=nrow(exp),dimnames=dimnames)

rt=avereps(rt)

#normalize

pdf(file="rawBox.pdf")

boxplot(rt,col = "blue",xaxt = "n",outline = F)

dev.off()

rt=normalizeBetweenArrays(as.matrix(rt))

pdf(file="normalBox.pdf")

boxplot(rt,col = "red",xaxt = "n",outline = F)

dev.off()

#differential

modType=c(rep("health",42),rep("sepsis",479))

design <- model.matrix(~0+factor(modType))

colnames(design) <- c("con","treat")

fit <- lmFit(rt,design)

cont.matrix<-makeContrasts(treat-con,levels=design)

fit2 <- contrasts.fit(fit, cont.matrix)

fit2 <- eBayes(fit2)

allDiff=topTable(fit2,adjust='fdr',number=200000)

write.table(allDiff,file="limmaTab.xls",sep="\t",quote=F,row.names=T)

write.table(allDiff,"limmaTab.txt",sep="\t",quote=F,row.names=T)

#write table

diffSig <- allDiff[with(allDiff, (abs(logFC)>logFoldChange & adj.P.Val < adjustP )), ]

write.table(diffSig,file="diff.xls",sep="\t",quote=F,row.names=T)

diffUp <- allDiff[with(allDiff, (logFC>logFoldChange & adj.P.Val < adjustP )), ]

write.table(diffUp,file="up.xls",sep="\t",quote=F,row.names=F)

diffDown <- allDiff[with(allDiff, (logFC<(-logFoldChange) & adj.P.Val < adjustP )), ]

write.table(diffDown,file="down.xls",sep="\t",quote=F,row.names=T)

#write expression level of diff gene

hmExp=rt[as.vector(diffSig[,1]),]

diffExp=rbind(id=colnames(hmExp),hmExp)

write.table(rt,file="data.txt",sep="\t",quote=F,col.names=T)

#volcano

pdf(file="vol.pdf",width = 15,height = 8)

xMax=max(-log10(allDiff$adj.P.Val))

yMax=max(abs(allDiff$logFC))

plot(-log10(allDiff$adj.P.Val), allDiff$logFC, xlab="-log10(adj.P.Val)",ylab="logFC",

main="Volcano", xlim=c(0,xMax),ylim=c(-yMax,yMax),yaxs="i",pch=20, cex=0.8)

diffSub=subset(allDiff, adj.P.Val<adjustP & logFC>logFoldChange)

points(-log10(diffSub$adj.P.Val), diffSub$logFC, pch=20, col="red",cex=2)

diffSub=subset(allDiff, adj.P.Val<adjustP & logFC<(-logFoldChange))

points(-log10(diffSub$adj.P.Val), diffSub$logFC, pch=20, col="green",cex=2)

abline(h=0,lty=2,lwd=3)

dev.off()

##The code of GSVA

setwd("C:\\Users\\lexb4\\Desktop\\GSVA\\03.GSVA")

inputFile="input.txt"

gmtFile="c2.cp.kegg.v6.2.symbols.gmt"

library(GSVA)

library(limma)

library(GSEABase)

rt=read.table(inputFile,sep="\t",header=T,check.names=F)

rt=as.matrix(rt)

rownames(rt)=rt[,1]

exp=rt[,2:ncol(rt)]

dimnames=list(rownames(exp),colnames(exp))

mat=matrix(as.numeric(as.matrix(exp)),nrow=nrow(exp),dimnames=dimnames)

mat=avereps(mat)

mat=normalizeBetweenArrays(mat)

c3gsc2=getGmt( gmtFile,

collectionType=BroadCollection(category="c3"),

geneIdType=SymbolIdentifier())

gsvaOut=gsva(mat,

c3gsc2,

min.sz=10,

max.sz=500,

verbose=TRUE,

parallel.sz=1)

gsvaOut=rbind(id=colnames(gsvaOut),gsvaOut)

write.table(gsvaOut,file="gsvaOut.txt",sep="\t",quote=F,col.names=F)

##The code of CIBERSORT

setwd("C:\\Users\\lexb4\\Desktop\\GEOimmune\\06.CIBERSORT")

source("GEOimmune.CIBERSORT.R")

results=CIBERSORT("ref.txt", "normalize.txt", perm=100, QN=TRUE)

##The code of univariate Cox regression

setwd("D:\\immunity\\COX3\\6_univariateCox")

outTab=data.frame()

library(survival)

rt=read.table("clinicalExp.txt",header=T,sep="\t",row.names=1,check.names=F)

rt1=log2(rt[,3:ncol(rt)]+1)

rt=cbind(rt[,1:2],rt1)

for(i in colnames(rt[,3:ncol(rt)])){

cox <- coxph(Surv(futime, fustat) ~ rt[,i], data = rt)

coxSummary = summary(cox)

outTab=rbind(outTab,cbind(gene=i,HR=coxSummary$coefficients[,"exp(coef)"],

z=coxSummary$coefficients[,"z"],

pvalue=coxSummary$coefficients[,"Pr(>|z|)"]))

}

write.table(outTab,file="univariateCox.xls",sep="\t",row.names=F,quote=F)

##The code of LASSO regression

library("glmnet")

library("survival")

setwd("D:\\ai\\cox2\\10.lasso")

rt=read.table("lassoInput.txt",header=T,sep="\t",row.names=1)

x=as.matrix(rt[,c(3:ncol(rt))])

y=data.matrix(Surv(rt$futime,rt$fustat))

fit <- glmnet(x, y, family = "cox", maxit = 10000)

pdf("lambda.pdf")

plot(fit, xvar = "lambda", label = TRUE)

dev.off()

cvfit <- cv.glmnet(x, y, family="cox", maxit = 10000)

pdf("cvfit.pdf")

plot(cvfit)

abline(v=log(c(cvfit$lambda.min,cvfit$lambda.1se)),lty="dashed")

dev.off()

coef <- coef(fit, s = cvfit$lambda.min)

index <- which(coef != 0)

actCoef <- coef[index]

lassoGene=row.names(coef)[index]

write.table(lassoGene,file="lassoGene.txt",sep="\t",quote=F,row.names=F,col.names=F)

##The code of multi-Cox regression

library(survival)

library(survminer)

setwd("D:\\ai\\cox2\\12.multiCox")

rt=read.table("multiInput.txt",header=T,sep="\t",check.names=F,row.names=1)

rt[,"futime"]=rt[,"futime"]

multiCox=coxph(Surv(futime, fustat) ~ ., data = rt)

multiCox=step(multiCox,direction = "both")

multiCoxSum=summary(multiCox)

outTab=data.frame()

outTab=cbind(

coef=multiCoxSum$coefficients[,"coef"],

HR=multiCoxSum$conf.int[,"exp(coef)"],

HR.95L=multiCoxSum$conf.int[,"lower .95"],

HR.95H=multiCoxSum$conf.int[,"upper .95"],

pvalue=multiCoxSum$coefficients[,"Pr(>|z|)"])

outTab=cbind(id=row.names(outTab),outTab)

write.table(outTab,file="multiCox.xls",sep="\t",row.names=F,quote=F)

pdf(file="forest.pdf",

width = 8,

height = 9,

)

ggforest(multiCox,

main = "Hazard ratio",

cpositions = c(0.02,0.22, 0.4),

fontsize = 0.7,

refLabel = "reference",

noDigits = 2)

dev.off()

riskScore=predict(multiCox,type="risk",newdata=rt)

coxGene=rownames(multiCoxSum$coefficients)

coxGene=gsub("`","",coxGene)

outCol=c("futime","fustat",coxGene)

risk=as.vector(ifelse(riskScore>median(riskScore),"high","low"))

write.table(cbind(id=rownames(cbind(rt[,outCol],riskScore,risk)),cbind(rt[,outCol],riskScore,risk)),

file="risk.txt",

sep="\t",

quote=F,

row.names=F)

##The code of ROC

library(pROC)

inputFile="input.txt"

outFile="ROC.pdf"

setwd("D:\\combined model\\our data\\41.multiVarROC")

rt=read.table(inputFile,header=T,sep="\t",check.names=F,row.names=1)

y=colnames(rt)[1]

bioCol=c("red","blue","green","yellow")

if(ncol(rt)>4){

bioCol=rainbow(ncol(rt))}

pdf(file=outFile,width=5,height=5)

roc1=roc(rt[,y], as.vector(rt[,2]))

aucText=c( paste0(colnames(rt)[2],", AUC=",sprintf("%0.3f",auc(roc1))) )

plot(roc1, col=bioCol[1])

for(i in 3:ncol(rt)){

roc1=roc(rt[,y], as.vector(rt[,i]))

lines(roc1, col=bioCol[i-1])

aucText=c(aucText, paste0(colnames(rt)[i],", AUC=",sprintf("%0.3f",auc(roc1))) )

}

legend("bottomright", aucText,lwd=1,bty="n",col=bioCol[1:(ncol(rt)-1)])

dev.off()

**Datasets description**

Two mRNA datasets (GSE65682 and GSE63042) of patients with sepsis from GEO public database were finally enrolled in the current study, with link as follow:

<https://www.ncbi.nlm.nih.gov/geo/query/acc.cgi?acc=GSE65682>

<https://www.ncbi.nlm.nih.gov/geo/query/acc.cgi>

The peripheral blood of septic patients was measured through mRNA expression microarrays in the two datasets. The blood were collected within 48 hours after ICU admission.

The dataset of training cohort was uploaded by Scicluna et al. from University Medical Center in Utrecht and the Academic Medical Center in Amsterdam. This dataset included 479 patients with sepsis and relatively completed prognostic data. In addition, 42 healthy participants (median age 35 years [IQR 30–63]; 24 [57%] of 42 were men) were also enrolled in the dataset of GSE65682.

Data of GSE63042 was uploaded by Langley et al. from Immunology department in University of New Mexico, which included 106 septic patients with 28-day mortality information.
